# Functional and Morphological Differences in the Play Face and Full Play Face in Lowland Gorillas, a Hominid Species: Implications for the Evolutionary Roots of Smile and Laugh Face

**DOI:** 10.1002/ajpa.70061

**Published:** 2025-05-08

**Authors:** Giada Cordoni, Martina Brescini, Luca Pirarba, Florinda Giaretto, Ivan Norscia

**Affiliations:** ^1^ Department of Life Sciences and System Biology University of Torino Turin Italy

**Keywords:** human laugh face, human smile, play asymmetry, play variability, Rapid Facial Mimicry

## Abstract

**Objective:**

Play Face (PF) and Full Play Face (FPF) in the great apes—homologous to human smile and laugh‐face—have been considered a single phenomenon. However, if natural selection has preserved two expressions, probably their adaptive value differs.

**Materials and Methods:**

We collected video data on play interactions in two lowland gorilla groups (*N* = 21; *Gorilla gorilla gorilla* ) housed at La Vallée des Singes and the ZooParc de Beauval (France). Lacking a tool tailored for gorillas during this study, we analyzed facial action‐unit activation via chimpFACS and OpenFace.

**Results:**

We found that PF and FPF activated partly different action units as it occurs for chimpanzees and humans' PF/FPF. We detected the rapid replication (Rapid Facial Mimicry [RFM]) of either PF or FPF that was associated with longer play sessions. Not‐mimicked PF was linked to increased play session variability (different types of play patterns) measured via the Shannon Index, whereas not‐mimicked FPF was associated with increased play asymmetry (imbalance between offensive/defensive patterns) measured via the Play Asymmetry Index.

**Discussion:**

Lowland gorillas may use PF to manage sessions that are more complex in terms of pattern types and FPF—a more salient signal—to prevent misunderstandings when the session is imbalanced. RFM of both expressions may favor the prolongation of play sessions by increasing player synchronization and possibly emotional sharing. Our study opens the door to further comparative studies on playful expressions in humans and other primates as a way to fine‐tune possible emotional communication and delineate potential evolutionary roots of Hominidae facial communication.

## Graphical abstract

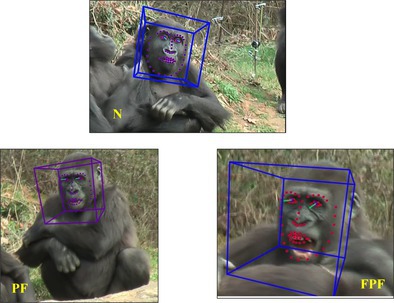

## 1 Introduction

Smiling, as we shall see, graduates into laughter. (Charles Darwin, *The Expression of the Emotions in Man and Animals*)

Facial expressions—visible facial movements associated with species‐specific behavioral repertoire that have a communicative meaning to group members (Waller et al. 2020)—are not a unique trait of humans. Other mammals show similar behavioral phenomena in terms of form and function, thus indicating that facial expressions have evolved long before the appearance of modern humans (Waller and Micheletta 2013).

In *Homo sapiens* , smile and laughter occupy a central role in social cohesion, and they have probably evolved in the social context of play (Provine 2000; Dezecache and Dunbar 2012; Bryant et al. 2016) where human and non‐human primates show common anatomical and functional features in facial behaviors (Cordoni and Palagi 2011; Davila‐Ross and Palagi 2022). Different variants of more or less genuine smiles and laughter exist in humans (Ambadar et al. 2009; Mascaró et al. 2021). Spontaneous smile and the visual component of laughter (hereafter, laugh face) share similar neural activation (mainly in the bilateral supplementary motor area; Iwase et al. 2002) and basic morphological features involving the contraction of face muscles such as *zygomaticus major* (lip corner pulling back and upwards), *orbicularis oculis* (crow's feet), and *orbicularis oris* (lip pressing; Parr et al. 2007; Caeiro et al. 2013). However, smile and laugh face do not completely overlap.

Human spontaneous smile and laugh face seem to be homologous to the relaxed open mouth expressions observed almost exclusively during play in non‐human primates, that is, the Play Face (PF) and the Full Play Face (FPF), respectively (van Hooff and Preuschoft 2003; Davila‐Ross and Dezecache 2021). The PF—that is almost present in all primate species (Palagi, Burghardt, et al. 2016)—usually involves the exposure of lower teeth, whereas during FPF—that has a more patchy distribution than PF within primates (Palagi, Burghardt, et al. 2016)—also the upper teeth are usually exposed due to the *levator labii superioris* muscle contraction (Davila‐Ross et al. 2015; Waller et al. 2020). In chimpanzees ( *Pan troglodytes* )—as in humans—both PF and FPF involve the contraction of the *zygomaticus major* (Parr et al. 2007; Waller et al. 2015, 2020). Furthermore, during FPF, chimpanzees, like humans, can exhibit parting lips, pulling lips back and upwards, jaw dropping, upper lip raising, and sometimes cheek raising (Davila‐Ross et al. 2015). It is worth noting that the activation levels of the different facial muscle action units (AUs) involved in the performance of both PF and FPF can show a certain degree of variability, highlighting that the two expressions represent a gradient of intensity. For example, in sanctuary chimpanzees, Davila‐Ross et al. (2015) found that 19% of PFs showed the activation of muscles linked to the raising of the upper lip, while Parr et al. (2007) did not find such activation in the Yerkes chimpanzees. For this reason, not only the exposure of the upper teeth but also the size of the mouth opening may account for the difference between PF and FPF (Davila‐Ross et al. 2015).

The automatic detection and analysis of facial AUs is one of the principal means for identifying and analyzing different facial expressions. Through specific toolkits (e.g., Facial Action Coding System [FACS]: Ekman and Friesen 1978; Ekman et al. 2002; OpenFace: Baltrušaitis et al. 2018), changes in facial muscle contraction related to different expressions have been identified in humans (Gilbert et al. 2021; Jeganathan et al. 2022), chimpanzees (Vick et al. 2007), rhesus macaques (Parr et al. 2010), gibbons (Waller et al. 2012), orangutans (Caeiro et al. 2013), dogs (Waller et al. 2013), cats (Caeiro et al. 2017), and horses (Wathan et al. 2015).

According to the *Complexity and Continuity Hypothesis* (Davila‐Ross and Dezecache 2021), the strong similarities in morphology and context point toward an evolutionary continuity, with no major changes, from non‐human hominids to modern humans.

During playful interactions, the primary function of smile/laugh face and PF/FPF is to signal the non‐aggressive behavioral intentions of the agent, although with different emotional grades (Pellis and Pellis 1996; Gervais and Wilson 2005; Cordoni and Palagi 2011, 2012; Palagi et al. 2022). This is particularly important during play‐fighting, a form of play where many behavioral patterns are drawn from aggressive context, and physical contact between players can be sustained for an extended period (Paquette 1994; Palagi, Burghardt, et al. 2016; Pellis and Pellis 2017; Cordoni et al. 2023). The *Power Asymmetry Hypothesis* (PAH, Preuschoft and van Hooff 1997) assumes that primate species living in despotic societies characterized by highly asymmetric hierarchical relationships need to produce unambiguous and distinct signals to distinguish play (especially, play‐fighting) from other interactions such as appeasement or affiliation. Although PAH was originally framed to explain between‐species variation in patterns of affiliative/appeasement/playful displays, it has been suggested that it could also be applied within species that show a large variation in dominance relationships between groupmates (Mehu and Dunbar 2008). During playful interactions, an asymmetry between players in behavioral pattern exchange and winner position attainment can occur (Bauer and Smuts 2007; Pellis et al. 2010; Pellis and Pellis 2017). For example, in chimpanzees, an increase in the use of playful expressions was associated with an increase in play asymmetry with the possible aim of reducing the misunderstanding between players when play becomes more competitive (Cordoni and Palagi 2011).

In humans, smile and laugh face may convey signals with different intensities (Redican 1982). However, smile is not only a sign of happiness (Ekman 2003). Depending on circumstances, smile can indicate nervousness, need to please, embarrassment, welcoming attitude, and these different meanings can be related to specific variations in smile morphological and dynamic features (Ambadar et al. 2009). On the other hand, laugh face is not exclusively considered an expression of humor or happiness. Indeed, laugh face with its auditory component can be defined as a social behavior that concurs in establishing and regulating social bonds and in reducing social tension (Scott et al. 2014; Wood and Niedenthal 2018; Palagi et al. 2022).

In non‐human primates, PF and FPF partly share similar morphology and functions (e.g., prolonging the session, avoiding escalation into aggression) and are often collapsed into a single phenomenon (Pellis and Pellis 1996, 1997; Palagi et al. 2007; Demuru et al. 2015). However, in different primate species (and all hominid species) evolution has maintained both PF and FPF signals (Davila‐Ross and Dezecache 2021). Furthermore, during play both PF and FPF can also be found separately (Waller and Cherry 2012; Palagi, Norscia, et al. 2019). Hence, from an adaptive standpoint, it is reasonable to hypothesize that these two signals may not have fully overlapping functions. For example, compared to PF, FPF has been found especially associated with higher intensity play in gorillas (Waller and Cherry 2012). According to the literature, FPF may be especially used: (i) in more tolerant societies where it is less likely that it is mistaken for a threat (in despotic groups such as those of some macaque species—for example, rhesus macaques—or baboons, the exposure of upper teeth may cause aggression by dominants; Thierry et al. 1989; van Hooff and Preuschoft 2003); (ii) when play is rough and requires the frequent display of salient play signals to communicate the non‐aggressive intent of players (Palagi, Burghardt, et al. 2016; Palagi, Cordoni, et al. 2016). In this respect, in lowland gorillas, play may escalate into aggression without salient play signals (Palagi et al. 2007; Palagi, Marchi, et al. 2019; Bresciani et al. 2021). Indeed, lowland gorillas ( *Gorilla gorilla gorilla* ) can use more FPF than chimpanzees possibly in relation to particularly rough play sessions (Palagi, Norscia, et al. 2019), although no study has clearly demonstrated this point.

Both smiles and laughs face in humans and PF/FPF in great apes can act as releasing stimuli (*sensu* Tinbergen 1952) and elicit the same facial expression in other interacting subjects (see for review Palagi et al. 2020). When the replication of the same facial expression occurs within 1 s from the stimulus emission, the phenomenon is known as Rapid Facial Mimicry (RFM; Dimberg and Thunberg 1998; Sestito et al. 2013). The facial replication is involuntary and automatic (as suggested by the response speed < 1 s) and occurs significantly more when the first stimulus (i.e., expression) is perceived by the potential receiver compared to when it is not perceived. Yet the difference between yes‐perception and no‐perception (of the first stimulus) conditions indicates that the phenomenon is mimicry and not simple synchronization (Palagi et al. 2020). RFM does not invoke advanced cognitive processes but rather basic automatic and involuntary processes by finding its roots in the automatic coupling of perception and action within the brain's sensorimotor areas, as foreseen by the Perception Action Model possibly involving the mirror neuron system (Gallese et al. 1996; Ferrari et al. 2003; de Waal and Preston 2017).

RFM is present during play in both human and non‐human animals showing open mouth displays (see review Palagi et al. 2020). In many primate and non‐primate species, the occurrence of RFM—compared to the simple presence of non‐replicated PF—is associated with a longer duration of playful interaction, thus a higher success of it (Tonkean macaques, Scopa and Palagi 2016; geladas, Mancini et al. 2013; lowland gorillas, Bresciani et al. 2021; chimpanzees, Palagi, Norscia, et al. 2019; orangutans, Davila‐Ross et al. 2008; dogs, Palagi et al. 2015; meerkats, Palagi, Marchi, et al. 2019).

In lowland gorillas—although with exceptions (Cordoni et al. 2022)—play is scarcely retained in adulthood (Masi et al. 2009; Cordoni et al. 2018). Gorillas show both PF and FPF (Palagi et al. 2007; Bresciani et al. 2021) and the occurrence of RFM—only verified by conflating PF and FPF—has been demonstrated during their social play sessions (Palagi, Norscia, et al. 2019; Bresciani et al. 2021). Furthermore, gorillas have a great number of facial muscles like the other Hominoidea (e.g., zygomaticus major, orbicularis oculi, levator labii superioris; Diogo et al. 2009, 2010). Hence, gorillas are particularly suitable to investigate the evolutionary basis of the differences between smile and laugh face. To this purpose, we investigated in lowland gorillas the differences in terms of morphology (i.e., facial muscle AU activation) and function between PF and FPF and their rapid replication (RFM). Based on the previous framework, we formulated the following predictions.

### 1.1 Prediction 1

If PF and FPF are basic expressions that have been preserved in the course of the evolution of primate facial expressions (Waller et al. 2020), we expect that—by applying the tool used for the expression analysis in other hominids (humans–OpenFace, chimpanzees–ChimpFACS)—we would detect with minimal error the activation of the same facial muscle units that activate in human and chimpanzee PF and FPF (*Prediction 1a*). Moreover, as PF and FPF should not possess the same exact morphology, we expect that the facial units that activate during the two expressions are not fully overlapping (*Prediction 1b*).

### 1.2 Prediction 2

As RFM is present in gorillas (Palagi, Norscia, et al. 2019; Bresciani et al. 2021) and PF and FPF can occur separately during play, we expected to find RFM of either PF or FPF (*Prediction 2a*). Furthermore, we expect that the duration of the playful session may be greater in the presence of RFM of either PF or FPF than in the presence of unreplicated facial signals or no signal (*Prediction 2b*).

### 1.3 Prediction 3

In gorillas, play is highly competitive and asymmetric, especially between juvenile/adolescent males (Palagi et al. 2007; Cordoni et al. 2018). In this light, we expected that FPF—more of a salient signal than PF (Palagi and Mancini 2011; Palagi, Burghardt, et al. 2016)—may be associated with high levels of play asymmetry to convey a clearer statement of positive mood by the agent (*Prediction 3a*). Furthermore, we also expected that in the case of more symmetric interaction, a less evident signal such as PF rather than FPF may be associated with an increased play variability (i.e., different types of playful behavioral patterns performed within a session; *Prediction 3b*).

## 2 Materials and Methods

### 2.1 Ethical Statement

The current study was purely observational and non‐manipulative; thus, approval was not required by the authors' institutional animal care committees.

### 2.2 Study Groups

The study was carried out on two family groups of lowland gorillas ( *Gorilla gorilla gorilla* ) housed at La Vallee des Singes (Romagne, France; hereafter, VDS) and the ZooParc de Beauval (Saint Aignan sur Cher, France; hereafter, BEA).

The VDS group was composed of 10 individuals (mean age ± SE: VDS 19.2 ± 5.10 Table 1). Two adolescent males (Mawete and Djomo) were castrated. Gorillas were fed outdoors with fruit, vegetables, seeds, leaves, and trunks five times per day during spring/summer and twice per day starting from September.

**TABLE 1:** The composition of the lowland gorilla family groups hosted at La Vallée des Singes (VDS) and the ZooParc de Beauval (BEA).

Subject	Sex	Age class and year of birth	Age (years)^a^	Kinship	Group	Total hours of observation	Total number of play sessions	Total number of PF	Total number of FPF
Yaoundé (YA)	M	Ad—1983	37	Father of all SubAd and Inf	VDS	92	9	6	14
Hakuna (HA)	F	Ad—1996	24	IV mother	VDS	80.5	24	7	3
Virunga (VI)	F	Ad—1970	50	No offspring	VDS	78	2	1	0
Moseka (MO)	F	Ad—1984	35	MW, DJ, and KO mother	VDS	71.5	0	0	0
Mahmah (MA)	F	Ad—2002	18	BA mother	VDS	79	85	48	53
Mawete (MW)	M	SubAd—2011	9	DJ and KO brother	VDS	62.5	209	70	209
Djomo (DJ)	M	SubAd—2008	12	MW and KO brother	VDS	63	43	15	50
Kouam (KO)	M	Inf—2016	4	MW and DJ brother	VDS	63	281	70	207
Ivindo (IV)	F	Inf—2017	3	—	VDS	70	190	32	114
Basoko (BA)	M	Inf—2020	0	—	VDS	75	86	49	112
Asato (AS)	M	Ad—1991	30	Father of all SubAd and Inf	BEA	43.5	0	0	0
Kabinda (KA)	F	Ad—1982	39	Mother YA, KO, MY, MA	BEA	42	0	0	0
Inge (IN)	F	Ad—1980	41	Mother KI	BEA	43	2	1	0
Sheila (SH)	F	Ad—1991	30	Mother MB, SA	BEA	42	0	0	0
Mapenzi (MA)	M	SubAd −2010	11	Siblings MY, KO, YA	BEA	41	8	1	2
Sawa (SA)	F	SubAd—2011	10	Siblings MB	BEA	42	3	0	0
Mayelè (MY)	F	SubAd—2013	8	Siblings YA, KO, MA	BEA	41.5	2	1	1
Yamba (YA)	M	SubAd—2015	6	Siblings MA, MY, KO	BEA	42.5	18	2	10
Kivano (KI)	M	SubAd—2016	5	—^b^	BEA	42	13	1	2
Mbaku (MB)	M	Inf—2018	3	Siblings SA	BEA	43	28	7	35
Kovanga (KO)	M	Inf—2019	2	Siblings MA, MY, YA	BEA	42.5	23	6	19

*Note:* The total hours of observation and the total number of play sessions in which each individual was involved and the total number of both PFs and FPFs performed by each individual were reported in the table.

Abbreviations: Ad, adult (female > 8 years; male > 12 years); F, female; Inf, infant (0–4 years); M, male; SubAd, sub‐adult (female 6–8 years; male 6–12 years).

^a^ years of age at the time of the study.

^b^ Kivano has no related individuals within the colony.

The BEA group comprised 11 individuals (mean age ± SE: 16.82 ± 4.52; Table 1). Two immature males (Mapenzi and Yamba) were castrated. The animals received food (fruit, vegetables, seeds, leaves, and trunks) six times per day.

VDS and BEA groups were managed in similar enclosures composed of both an indoor (VDS 150 m^2^, BEA 200 m^2^) and outdoor (a wooded island surrounded by a water canal; VDS 3400 m^2^, BEA 2000 m^2^) facilities. The enclosures were enriched with trees, lianas, trunks, straw, and platforms. During the day, gorillas can move freely between the indoor and outdoor facilities and can socially interact. In both groups, silverbacks were the fathers of immature subjects, and all adult females were treated with oral contraceptives.

### 2.3 Data Collection

We collected video data on both colonies during the following periods: August–October 2020/March–June 2021/March–June 2022 for VDS and June–October 2021 for BEA. For video recording, a full HD camera (Panasonic HDC‐SD9) was used.

The observers (L.P., F.G.) were trained by G.C. in animal recognition and the application of methodological procedures (i.e., scan and all‐occurrences sampling methods; see below). Additionally, before starting the video analysis independently, the observers received training from G.C. in identifying behavioral patterns (see Table 2) and distinguishing between PF and FPF. Ten percent of the recorded play sessions (approximately 100 sessions) were analyzed—either in slow motion or frame‐by‐frame using the freeware Avidemux 2.7.8—by both observers simultaneously and separately. Their agreement in identifying behavioral patterns and distinguishing between PF and FPF was assessed using Cohen's kappa, which indicates the proportion of agreement beyond what would be expected by chance. Interobserver reliability between the video coders was calculated using the R function “cohen.kappa” and the “irr” and “psych” libraries (R version 3.5.3). Training was concluded when the interobserver reliability reached a Cohen's kappa value of 0.80 for PF and FPF and from 0.60 to 0.94 for playful patterns recorded in this study (see Table 2).

**TABLE 2:** Play and aggressive behavioral patterns of lowland gorillas considered in this study (see the text for definitions of these three categories).

Play patterns	Definition
Attempt play bite^a^	The gorilla unsuccessfully tries to close her/his mouth on the partner's body
Peek a boo^a^	The gorilla hides and suddenly pops out from a shelter
Play bite^a^	The gorilla closes her/his mouth on the partner's body in a non‐harmful way
Play brusque rush^a^	The gorilla jumps with her/his four limbs on the playmate generally in a quadrupedal position and either bounces away or stays
Play chase^a^	The gorilla runs behind the playmate (social play) by often changing her/his direction
Play climb or stand on another^a^	The gorilla climbs or stands on the playmate's body independently of the position of the playmate (sitting, lying or standing)
Play drag^a^	The gorilla hauls the playmate taking her/him from the limbs
Play eye cover^a^	The gorilla covers the eyes of the playmate
Play jump^a^, ^b^	The gorilla gently jumps alone (in this case the pattern is considered neutral) or on the playmate (in this case the pattern is considered offensives) only with feet generally in a quite bipedal position. Play jumps are generally small, mainly stationary, with little or no moving forward
Play kick^a^	The gorilla gently uses her/his feet to hit the playmate
Play pull^a^	The gorilla moves the playmate towards her/him with hands/feet during play
Play push^a^	The gorilla displaces the playmate far from her/him with hands/feet
Play retrieve^a^	The gorilla blocks with her/his hands the playmate to prevent her/his flight. It is different from play pull that is generally performed with both feet and hands during play
Play slap^a^	The gorilla uses her/his open hands for hitting any part of the playmate's body
Play stamp^a^	The gorilla hits on the ground or on the playmate with her/his feet in a repeated way
Play tug‐of‐war^a^	The gorillas contend an object and pull it toward themselves
Rough and tumble^a^	The gorillas play in tight and continuous physical contact by employing many of patterns described in this table (e.g., bite, kick, slap, stamp)
Play flee^c^	The gorilla runs far from the partner which are chasing it
Play shelter^c^	The gorilla protects herself/himself from playmate slaps, bites, etc. by putting its arms over the head
Play wriggle^c^	The gorilla wriggles to get rid of the grip of the playmate
Acrobatic play^b^	The gorilla swings hanging/jumps from a support and makes somersaults/pirouettes in a solitary or social manner
Airplane^b^	The older gorilla holds the smaller playmate with hands/feet above her/his head while lying on the ground
Pirouetting^b^	The gorilla performs somersaults/pirouettes on herself/himself or hanging from a rope. Pirouetting can be a part of acrobatic play
Play carry^b^	The gorilla dorsally or ventrally carries the playmate (typical of play mothering). The carried subject lies down on the carrier’ dorso or he/she is in contact with the carrier's lower abdomen in a sort of embrace. The carrier moves.
Play “give me five”^b^	Two gorillas interact face‐to‐face and slap each other palms
Play grab^b^	The gorilla gently massages the playmate holding her/him tightly
Play manipulation^b^	The gorilla takes and explores an object without using it for any specific goal
Play moon walk^b^	The gorilla walks backward, generally keeping her/his eyes fixed on the playmate
Play pat^b^	The gorilla repeatedly and gently touches with the palm of her/his hand the partner's body
Play piggy back ride^b^	The gorilla is placed with a leg on each side of the back of the playmate and his/her torso is erected. This position looks like that of horse rider. The carrier can move or not.
Play roll^b^	The gorilla turns its body from side to side while supine
Play shake the rope^b^	The gorilla strongly moves the rope on which the playmate is hanging
Play slide down^b^	The gorilla slides down from hill, tree, rocks or other equipment
Play turn around^b^	The gorilla runs/walks alone around an object without changing her/his direction
Play walk^b^	The gorilla follows the playmate or goes back and forth
Somersault^b^	The gorilla flips over the ground or on vertical supports in solitary or social manner
Tickle^b^	The gorilla tickles with hands/feet any part of the partner's body
Play invitation	The gorilla performs one or more playful behavioral patterns for inviting the fellow to play
Full play face	The gorilla opens her/his mouth with both upper and lower teeth exposed
Play face	The gorilla opens her/his mouth with only the lower teeth exposed

^a^ Offensive pattern.

^b^ Neutral pattern.

^c^ Defensive pattern.

By scan animal sampling (Altmann 1974) we collected at 10‐min intervals the overall group daily activity by recording both solitary (moving, resting, foraging, feeding) and social (play, grooming, body contact, aggression, proximity) behaviors. Using this method, we collected a total of 254 h of observations. Specifically, a total of 60 out of the 254 h were spent by animals playing (60 h of play corresponding to 360 scans). We also employed all occurrences sampling method (Altmann 1974) for gathering data on social playful interactions: (i) players' identities, (ii) playful patterns in sequential order (Table 2), (iii) playful expressions (PF and FPF) and their durations, and (iv) play session duration. We created Excel sheets in which, for each playful session, we reported all identified play patterns (including PF and FPF) in their sequential order of occurrence as determined by video analysis. It is important to note that scans were taken while all occurrences sampling was going (a total of 254 h of all occurrences). We analyzed a total of 1026 play‐fighting sessions (the total number of play sessions in which each gorilla was involved in is reported in Table 1). All sessions involved physical contact between players and, therefore, did not differ in their intensity levels and play category. We recorded a total of 317 PFs and 831 FPFs (the total number of PFs and FPFs performed by each gorilla during their playful interactions is reported in Table 1).

### 2.4 Facial Unit Identification—FACS and OpenFace Systems

During the current study, in the absence of a tool specifically designed to detect the activation of facial muscle AUs in gorillas (GorillaFACS has been very recently implemented by Correia‐Caeiro et al. 2025), we employed both FACS adapted for chimpanzees (ChimpFACS; Vick et al. 2007) and OpenFace 2.0 (Ambadar et al. 2009; Baltrušaitis et al. 2018) for humans to identify which specific facial muscle AUs were activated during the performance of PF and FPF by gorillas. By using FACS, it is possible to compare facial behaviors independently of face morphology variability across individuals (e.g., bone structure, fatty deposit; Waller et al. 2007, 2008). FACS identifies the contraction (binary: 0 = no contraction, 1 = yes contraction) of 33 facial AUs and often identifies the contraction of a group of muscles instead of a single muscle.

OpenFace 2.0 can recognize facial expressions through detecting the facial AU activation (Amos et al. 2016). It can also estimate the intensity of 17 AUs (1, 2, 4, 5, 6, 7, 9, 10, 12, 14, 15, 17, 20, 23, 25, 26, and 45; Baltrušaitis et al. 2018). Since OpenFace uses a new Convolutional Neural Network‐based face detector and an optimized facial landmark detection algorithm, it is possible to identify AUs also when the face is non‐frontal or/and in low illumination conditions. The software extracts facial characteristics by using histograms of oriented gradients and reduces dimensionality by using PCA. A subject‐specific neutral expression is extracted by computing the median value of face descriptors in the video sequence, assuming that most frames contained neutral expressions. The extracted median face is subtracted from the feature descriptor, leading to a normalized feature. The normalized feature vector describes the dynamic change from the neutral expression. The AU recognition framework uses linear kernel support vector machines for AU occurrence detection and support vector regression for AU intensity estimation (Baltrušaitis et al. 2018).

**FIGURE 1:**
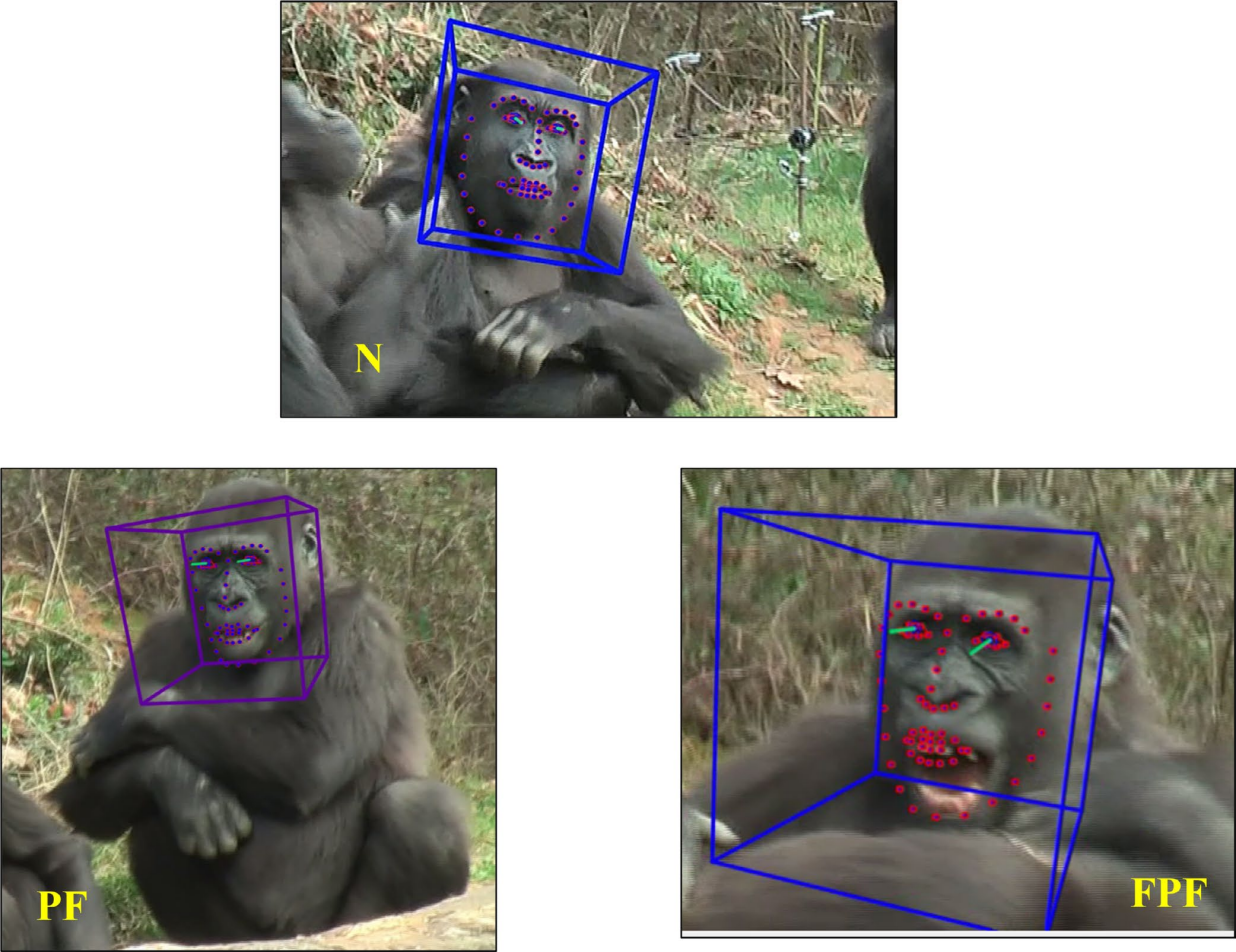
An example of image capturing with OpenFace 2.0. In the three images (N, PF, and FPF), 3D facial landmarks (red/blue dots), head pose traking (blue/violet 3D polygons) and eye gaze traking (light green lines) are represented. FPF, full play face; N, neutral face; PF, play face.

We applied both FACS and OpenFace to evaluate the AU activation of PFs (for eight selected gorillas) and FPFs (for nine selected gorillas; see Figure 1 and Video S1A,B). For each selected individual, at least one PF and one FPF were analyzed (see Table S2). Since FACS and OpenFace were not designed for gorillas, we selected the facial expressions (PF and FPF) that could be very clearly seen on videos and were mostly frontal for a preliminary assessment. It is important to note that the main purpose of this part of the research was to verify that PF and FPF were different, not to provide definitive and fine indications of all the AUs activated, especially since that would require a GorillaFACS, which is not yet available at the time of our study (Correia‐Caeiro et al. 2025). M.B. coded these expressions with both FACS (manuals for coding were used) and OpenFace and confirmed the types of PFs determined by the observers (L.P. and F.G.) before the coding with software. Then, I.N. and G.C. re‐coded the PFs and FPFs of the selected gorillas by FACS and OpenFace, respectively. The inter‐coder reliability reached a Cohen's kappa value equal to 0.81 (17 PFs and 15 FPFs were assessed).

### 2.5 Operational Definitions

#### 2.5.1 Play Session

A play session started when one individual directed any playful behavioral patterns (Table 2) toward a conspecific and ended when one of the players or a third uninvolved individual interrupted the interaction (Cordoni et al. 2021, 2022). The mean duration (in seconds) of the play session was 42.80 ± 2.10 SE, with a minimum value of 5.0 s and a maximum value of 405.0 s. We distinguished behaviors in offensive (*O*—behaviors of attack toward the partners), defensive (*D*—behaviors of avoidance/protection toward the attack by the partner), and neutral (*N*—neither offensive nor defensive behaviors; see Table 2; Cordoni et al. 2021, 2022, 2023).

#### 2.5.2 PF and FPF Duration

The duration of all PFs and FPFs observed in the collected videos was calculated by a frame‐by‐frame video analysis starting from the first frame in which the gorilla's lips were separated until the first frame in which the lips were closed again (Bertini et al. 2021; Bresciani et al. 2021). The inter‐observer reliability for the duration of the two types of playful expressions was assessed using the mean duration‐per‐occurrence inter‐observer agreement (IOA) for a high number of timings (Reed and Azulay 2010). We selected about 10% of PFs (durations of 40 PFs assessed) and FPFs (durations of 90 FPFs assessed) recorded on videos for calculating the IOA. The duration of each PF/FPF (i.e., duration measurement trial) was evaluated by the two observers separately. IOA was determined for each duration measurement trial by dividing the smaller duration by the higher duration reported by the two observers. For example, if the duration of a PF was 15 s for one observer and 20 s for the other observer, the IOA for this duration measurement trial was 15/20 = 0.75. IOA values of all duration measurement trials were summed and divided by the total number of trials; the result was transformed in %. The IOA for PF and FPF durations was equal to 93%.

#### 2.5.3 The Evaluation of the Presence of the RFM

**FIGURE 2:**
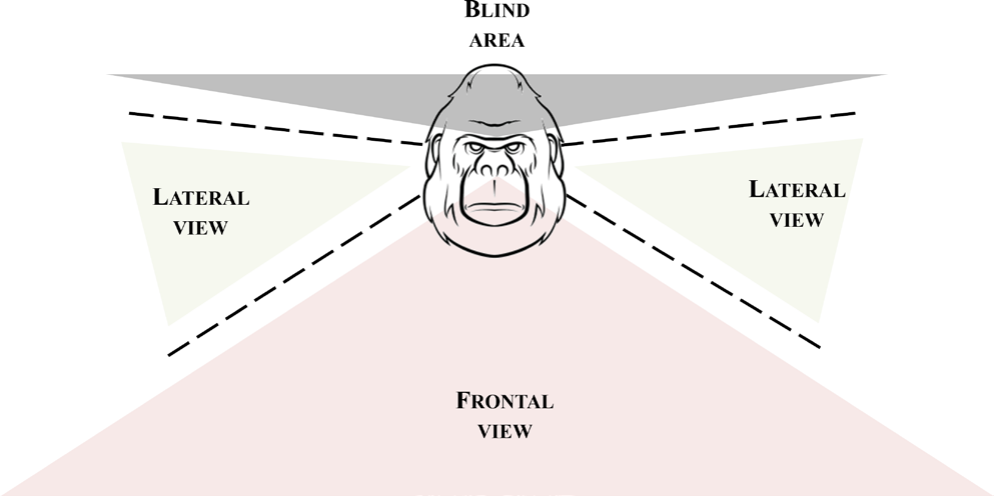
Graphical representation of the individual perception condition. In the *yes‐perception* condition the first stimulus by the trigger fell into frontal or lateral view of the potential receiver. In the *no‐perception* condition the stimulus fell into the blind area of the potential receiver.

To demonstrate the presence of RFM in the groups under study, each time a player (hereafter, the trigger) emitted the first PF/FPF (hereafter, first stimulus), we evaluated the presence or absence of a PF/FPF emitted by the play partner (hereafter, the potential responder) within 1 s after the emission of the first stimulus under two perception conditions (Figure 2): the first stimulus was perceived by the potential responder (i.e., the stimulus fell within the visual field of the responder; *yes‐perception* condition) and the first stimulus was not perceived by the potential responder (i.e., the stimulus did not fall within the visual field of the responder; *no‐perception* condition). All doubtful cases (e.g., when observers did not clearly see the faces of the players from the video, or the head positions of the players were not clear) were discarded from the analysis. The agreement between the two observers in determining both the *yes‐perception* and *no‐perception* condition was measured across 300 play sessions (approximately 30% of the total play sessions analyzed), including 15 gorillas for *yes‐/no‐perception* of PF and 12 gorillas for *yes‐/no‐perception* of FPF. Cohen's kappa reached a value of 0.82 for *yes‐/no‐perception* of PF and 0.83 for *yes‐/no‐perception* of FPF. We complemented this analysis with another that considered how many PFs/FPFs emitted by the trigger were perceived vs not perceived to the potential responder and how many of these PFs/FPFs were replicated or not replicated by the potential responder within 1 s after their emission. Based on the definition of RFM (see Section 1), all these analyses included only congruent responses by the potential responder to the first stimulus: a PF in response to a PF and a FPF in response to a FPF (exact facial matching).

We then compared the individual levels of PF_RFM with individual levels of FPF_RFM by dividing the number of PFs/FPFs replicated by the potential responder by the number of PFs/FPFs they perceived.

#### 2.5.4 Play Session Duration

To evaluate possible effects of PF, FPF, or RFM on play session duration, we considered five different conditions: (i) players performed at least two not‐mimicked PFs only (*PF‐not‐mim*); (ii) players performed at least two not‐mimicked FPFs only (*FPF‐not‐mim*); (iii) at least one event of RFM of PF (but not FPF) occurred (*PF_RFM*); (iv) at least one event of RFM of FPF (but not PF) occurred (*FPF_RFM*), and (v) at least one event of both RFM of PF and RFM of FPF occurred in the same play session (*PF&FPF_RFM*). For each condition, we determined the time period (in seconds) between the occurrence of the first PF, FPF, or RFM event and the end of the play session (i.e., the time period remaining from the first PF/FPF/RFM). Then, we compared these time periods across the five conditions. We excluded from the analysis those play sessions in which no PF/FPF or RFM events occurred, as well as ambiguous cases where not‐mimicked PF and FPF were both present in the same session. Furthermore, to determine whether RFM of either PF or FPF occurred near the start (for prolonging the playful session) or the end (for signaling the end of the playful session) of a play session, we calculated the halfway point of duration (in seconds) of each session. We then evaluated whether the first RFM event occurred before (near the session start) or after (near the session end) this midpoint (a binomial variable: before the midpoint = 1; after the midpoint = 0).

#### 2.5.5 Play Asymmetry Index (PAI)

PAI was calculated by considering the number of offensive and defensive behavioral patterns (see Table 2) exchanged between players on the total number of patterns (offensive + defensive + neutral) composing the session (Cordoni et al. 2018, 2023):

$$\mathrm{PAI}=\frac{\left({\text{offensive}}_{A\to B}+{\text{defensive}}_{B\to A}\right)-\left({\text{offensive}}_{B\to A}+{\text{defensive}}_{A\to B}\right)}{\left(\text{offensive}+\text{defensive}+\text{neutral}\right)}.$$

In the formula, A and B represent the players and the arrow (→) who directed behaviors to whom. PAI was calculated before and after a PF/FPF or RFM event (see Section 2.5.7) and ranged from −1 to +1. PAI values equal to or near −1 indicate a complete asymmetry in favor of Player B, who performed more offensive/fewer defensive patterns or received fewer offensive/more defensive patterns from Player A. PAI values equal to or near +1 indicate complete asymmetry in favor of Player A. PAI values equal to or near 0 indicate complete symmetry in the exchange of offensive and defensive behaviors between players.

#### 2.5.6 Shannon Index (*H*′)

The *H*′ (Shannon 1948; Keylock 2005) was an ecological index that was adapted for evaluating the level of play variability in terms of different types of behavioral patterns performed by players during a session (Cordoni et al. 2023). All offensive, defensive, and neutral patterns described in Table 2 were considered for the calculation of *H*′. The index was calculated as follows:

$$H^{\prime }\;=-\sum \;\left[{\left(n_{i}/N\right)}^{*}\;\left(\ln\;n_{i}/N\right)\right]$$

In the formula, *n*_*i*_ represented the number of behaviors belonging to the type *i* and *N* represented the total number of behaviors composing a session. For example, for a session composed by “play slapping—play slapping—play sheltering—play slapping—pirouetting,” *n*_*i*_ = 3 (behavioral types = *play slapping*, *play sheltering*, and *pirouetting*), *N* = 5 (total number of behavioral patterns composing the session that is 3 *play slapping* + 1 *play sheltering* + 1 *pirouetting*). A high value of *H*′ indicates a great behavioral pattern variability.

#### 2.5.7 Sequential Analysis

To evaluate possible variations in play asymmetry and variability within a session related to the occurrence of PF, FPF, or RFM, we conducted a sequential analysis on each play session, as detailed in the Excel sheets (see Data Collection). When a PF, FPF, or RFM (of both PF and FPF) event occurred within a session, we selected—if possible—the four play patterns before and the four play patterns after a playful expression (PF or FPF) or mimicry event (PF_RFM or FPF_RFM). Based on these patterns, we calculated the PAI and *H*′ values before and after a PF, FPF, or an RFM event. Consequently, for each PF, FPF, or RFM event within a session, we obtained PAI_before_, PAI_after_, *H*′_before_, and *H*′_after_. We selected four patterns before and after a playful expression or RFM event to standardize the data and give the probability to each player to perform at least one offensive and one defensive pattern. Furthermore, because there may be a session duration bias (high variability in session duration), we standardized these analyses by selecting four patterns before and four patterns after. When multiple playful expressions or RFM events occurred consecutively within a session, we calculated PAI_before_, PAI_after_, *H*′_before_, and *H*′_after_ only if the two expressions or RFM events were separated by at least four play patterns (excluding the four patterns used for index calculation). Thus, we excluded from the analysis consecutive playful expressions or RFM events separated by fewer than four patterns.

### 2.6 Statistical Analyses

The distribution of time periods in the five conditions (see Section 2.5) was not normal (Shapiro–Wilk test 0.643 ≤ *W* ≤ 0.862; 0.001 ≤ *p* ≤ 0.029); thus, we employed the non‐parametric Kruskal–Wallis test used for *k*‐independent samples. We also applied the Monte Carlo randomization (10.000 permutations) because of the non‐independence of data due to the fact that a same individual could be present in more than one dyad. In case of test significance, we applied a post hoc test with Bonferroni correction for pairwise comparison.

The median durations of PF/FPF, AU intensity values, levels of PF_RFM and FPF_RFM, and PAI/*H*′ values (Shapiro–Wilk test 0.612 ≤ *W* ≤ 0.800; 0.001 ≤ *p* ≤ 0.007) were compared at the individual level by employing the non‐parametric Wilcoxon exact test for two dependent samples with Bonferroni's correction when necessary.

We used a binomial test to evaluate whether the first RFM event within the session occurred more frequently before or after the halfway point of the duration (in seconds) of each session.

A 2 × 2 contingency chi‐square test was used to compare the number of PFs/FPFs replicated or not replicated within 1 s by the potential responder under perceived and not perceived conditions. To ensure more conservative results, Yates' Continuity Correction was applied.

To compare the individual number of PF/FPF performed per play session (Shapiro–Wilk test 0.146 ≤ *W* ≤ 0.183; 0.066 ≤ *p* ≤ 0.200), we employed the parametric paired *t*‐test for dependent samples.

To demonstrate the occurrence of RFM for both PF and FPF, we ran two generalized linear mixed models (GLMM). The first model (GLMM_PF_) included as the target variable the presence/absence (binomial variable; 0 = absence, 1 = presence) of a PF performed by the potential responder within 1 s after the emission of the first PF by the trigger. The fixed factors were the perception condition of the potential responder (binomial variable; 0 = *no‐perception*, 1 = *yes‐perception*) and the group (binomial variable; 1 = BEA group, 2 = VDS group). The dyad IDs (trigger‐potential responder) were entered as random factors.

The second model (GLMM_FPF_) included as a target variable the presence/absence (binomial variable; 0 = absence, 1 = presence) of a FPF performed by the potential responder within 1 s after the emission of the first FPF by the trigger. The fixed factors were the perception condition of the potential responder (binomial variable; 0 = *no‐perception*, 1 = *yes‐perception*) and the group (binomial variable; 1 = BEA group, 2 = VDS group). The dyad IDs (trigger‐potential responder) were entered as random factors.

## 3 Results

### 3.1 Preliminary Analyses

**FIGURE 3:**
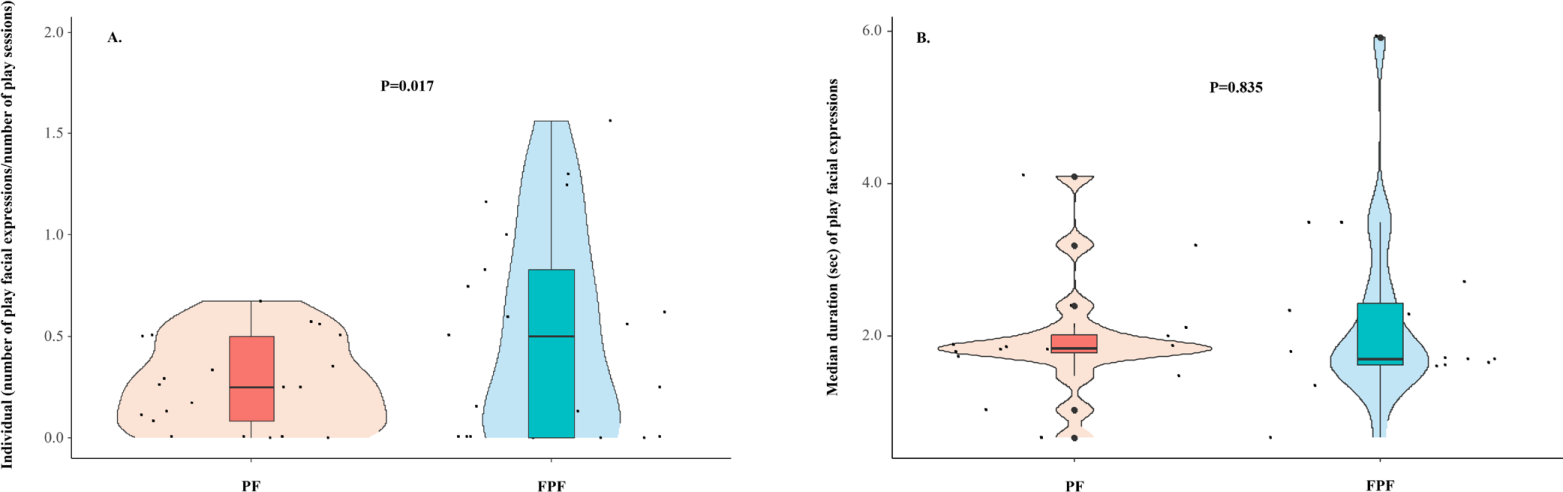
Violin plots with included boxplots representing (A) the levels of play face (PF) and full play face (FPF) calculated as the total number of PFs/FPFs performed by an individual divided by total number of play sessions in which this individual was involved, and (B) the median duration values (in seconds) of both PF and FPF performed by each individual. The shape of the violin represents the density estimate of the variable considered: the more data points in a specific range the larger the violin shape is for that range. Dots represent the individual data points (*N*_individuals_ = 21). In the middle of each density curve, there is a small boxplot with the rectangle showing the ends of the first and third quartiles and the central line showing the median value.

We carried out this analysis at the individual level by comparing the number of PFs/FPFs performed per play session (i.e., total number of PFs/FPFs performed by an individual divided by total number of play sessions in which this individual was involved). We found that levels of FPFs were higher than levels of PFs (paired *t*‐test *N*_individuals_ = 21, gl = 20, *t* = −2.608, *p* = 0.017; mean value of PFs/FPFs per session ± SE: PF 0.26 ± 0.05; FPF 0.51 ± 0.11; Figure 3A).

For each individual, we also calculated the medians of both PF and FPF durations. The median durations did not significantly differ between PF and FPF (Wilcoxon exact test *N*_individuals_ = 16, *T* = 56.0, ties = 1, *p* = 0.835; Figure 3B). The sample size is 16 because this analysis included only individuals for whom both PF and FPF were recorded (see Table 1).

The percentages of play sessions during which only one type of playful expression was performed were 3.1% and 5.5% for PF and FPF, respectively.

### 3.2 Prediction 1

Through the use of chimpFACS, during the performance of PFs we detected the activation of the AUs reported in Table S2 and Figure S3. We obtained 14 different configurations for PF and—even though the sample size is limited (*N* = 8 gorillas)—we found that four AUs were always or almost always present in the configurations detected: AU12 (*zygomaticus major*—lip corner puller), AU16 (*depressor labii inferioris*—lower lip depressor), AU25 (*depressor labii*, *orbicularis oris*—lips part), and AU26 (non‐mimetic muscle—jaw drop). We also reported in Table S2 and Figure S4 the activation of the AUs during the performance of FPFs (*N* = 9 gorillas). We obtained six different configurations for FPF and eight AUs were always or almost always present in the configurations detected: AU06 (*orbicularis oculi*, *pars orbitalis*—cheek raiser), AU09 (*levator labii superioris alaquae nasi*—nose wrinkler), AU10 (*levator labii superioris*—upper lip raiser), AU12, AU16, AU25, AU26, and AU27 (non‐mimetic muscle—mouth stretch). AU10 was activated only twice and AU27 only once during the performance of PFs, thus suggesting that PF and FPF may differ to a certain extent in upper lip raising and mouth stretch.

By OpenFace 2.0, we obtained the intensity values of the AUs involved in the performance of both PF and FPF (see raw data provided as Supporting Information). OpenFace does not estimate the intensity value of both AU16 and AU27; for this reason, the following analysis did not include these two AUs. For each AU, we compared the intensity values between PF and FPF at the individual level, and we obtained the following results (Bonferroni's correction *α* = 0.005): AU1—inner brow raiser (Wilcoxon exact test *N*_individuals_ = 8, *T* = 4, ties = 2, *p* = 0.219), AU2 outer brow raiser (*N*_individuals_ = 8, *T* = 0, ties = 0, *p* = 1.000), AU6 (*N*_individuals_ = 8, *T* = 10.5, ties = 0, *p* = 0.328), AU9 (*N*_individuals_ = 8, *T* = 5, ties = 3, *p* = 0.498), AU10 (*N*_individuals_ = 8, *T* = 0, ties = 0, *p* = 0.008), AU12 (*N*_individuals_ = 8, *T* = 4.5, ties = 0, *p* = 0.063), AU17—chin raiser (*N*_individuals_ = 8, *T* = 3, ties = 0, *p* = 0.039), AU 25 (*N*_individuals_ = 8, *T* = 10, ties = 0, *p* = 0.313), AU 26 (*N*_individuals_ = 8, *T* = 11, ties = 0, *p* = 0.383), and AU45—blink (*N*_individuals_ = 8, *T* = 13.5, ties = 1, *p* = 0.984). Although no comparison reached statistical significance, the probability of AU10 was the closest to significance when considering the Bonferroni's correction. We can infer that AU10 intensity tended to be higher during FPF than PF performance.

Hence, the results of both chimpFACS and human OpenFace suggested that PF and FPF may differ principally in the activation of AU10 (higher lip raising) and AU27 (mouth stretching) that are mainly responsible for the exposure of upper teeth.

### 3.3 Prediction 2

**FIGURE 4:**
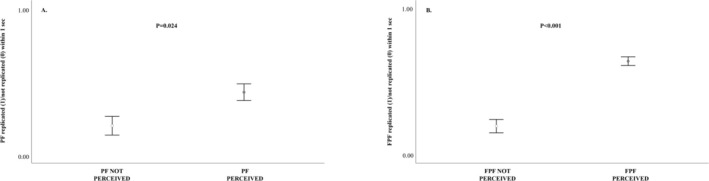
Error bars representing (A) the number of PFs replicated (= 1) and not replicated (= 0) by potential responders within 1 s after the emission of the first PFs by triggers under the two perception conditions: PF perceived (= 1) and PF not‐perceived (= 0); number of PF perceived and replicated = 33; number of PF perceived and not replicated = 44; number of PF not perceived and replicated = 8; number of PF not perceived and not replicated = 32; and (B) the number of FPFs replicated (= 1) and not replicated (= 0) by potential responders within 1 s after the emission of the first FPFs by triggers under the two perception conditions: FPF perceived (= 1) and FPF not‐perceived (= 0); number of FPF perceived and replicated = 171; number of FPF perceived and not replicated = 96; number of FPF not perceived and replicated = 15; number of FPF not perceived and not replicated = 61. The length of bars indicates how spread the data are around the mean value (colored dots).

*Prediction 2a*. We ran GLMM_PF_ to verify the occurrence of PF_RFM. The full model (including the fixed factors) and the null model (only including the random factors) significantly differed (likelihood ratio test: *χ* ^2^ = 8.281, df = 2, *p* = 0.016). Because the predictor had a significant effect on the target variable, we applied the drop1 procedure. We found that the perception condition significantly affected the target variable: the probability of a PF emission by the potential responder was higher when they visually perceived the PF emitted by the trigger (*yes‐perception* condition) compared to when they did not visually perceive (*no‐perception* condition) the first stimulus (Table 3). The 2 × 2 contingency chi‐square test revealed that PFs were significantly more replicated when they were perceived by the potential responder compared to the other conditions (*χ* ^2^ = 5.080, df = 1, *p* = 0.024; Figure 4A).

**TABLE 3:** Results of the GLMM_PF_ and GLMM_FPF_.

	Estimate	SE	*z*‐value	*p*
GLMM_PF_ target variable: presence/absence of a PF by the potential responder within 1 s after the first stimulus by the trigger (only congruent responses considered PF→PF) Random factors: dyad IDs (*N*_dyads_ = 117) full model versus null model: *χ* ^2^ = 8.281, gl = 2, *p* = 0.016				
Intercept	−2.373	0.854	−2.780	0.005
Perception condition (*yes‐perception*)	1.064	0.463	2.296	**0.022**
Group (VDS)	1.109	0.814	1.363	0.173
	Variance Inflation Factor (VIF)			
Perception condition	1.001			
Group	1.001			
GLMM_FPF_ target variable: presence/absence of a FPF by the potential responder within 1 s after the first stimulus by the trigger (only congruent responses considered FPF→FPF) Random factors: dyad IDs (*N*_dyads_ = 343) full model versus null model: *χ* ^2^ = 50.239, gl = 2, *p* < 0.001				
Intercept	−1.469	0.562	−2.615	0.009
Perception condition (*yes‐perception*)	2.117	0.338	6.259	**< 0.001**
Group (VDS)	−0.157	0.501	−0.313	0.755
	Variance Inflation Factor (VIF)			
Perception condition	1.003			
Group	1.003			

*Note:* Random factor = dyads composed of trigger and potential responder. Bold values indicate statistical significance (*p* < 0.05). The predictor was dummy‐coded, with the reference categories perception condition (*yes‐perception*) and group (VDS).

Abbreviations: SE, standard error; VDS, La Vallée des Singes.

GLMM_FPF_ was carried out to verify the occurrence of FPF_RFM. The full model and the null model significantly differed (likelihood ratio test: *χ* ^2^ = 50.239, df = 2, *p* < 0.001). We found that the perception condition significantly affected the target variable: the probability of a FPF emission by the potential responder was higher when they visually perceived the FPF emitted by the trigger (*yes‐perception* condition) compared to when they did not visually perceive (*no‐perception* condition) the first stimulus (Table 3). The 2 × 2 contingency chi‐square test showed that FPFs were significantly more replicated when they were perceived by the potential responder compared to the other conditions (*χ* ^2^ = 45.024, df = 1, *p* < 0.001; Figure 4B). Summing, the RFM was determined for both PF and FPF separately.

The levels of FPF_RFM tended to be higher than the levels of PF_RFM (Wilcoxon exact test *N* = 10, *T* = 7.0, ties = 1.0, *p* = 0.074; mean level of RFM ± SE: PF_RFM 0.31 ± 0.08; FPF_RFM 0.57 ± 0.10). The sample size was smaller (*N* = 10) because the analysis only included individuals who had both perceived and replicated a PF and a FPF.

**FIGURE 5:**
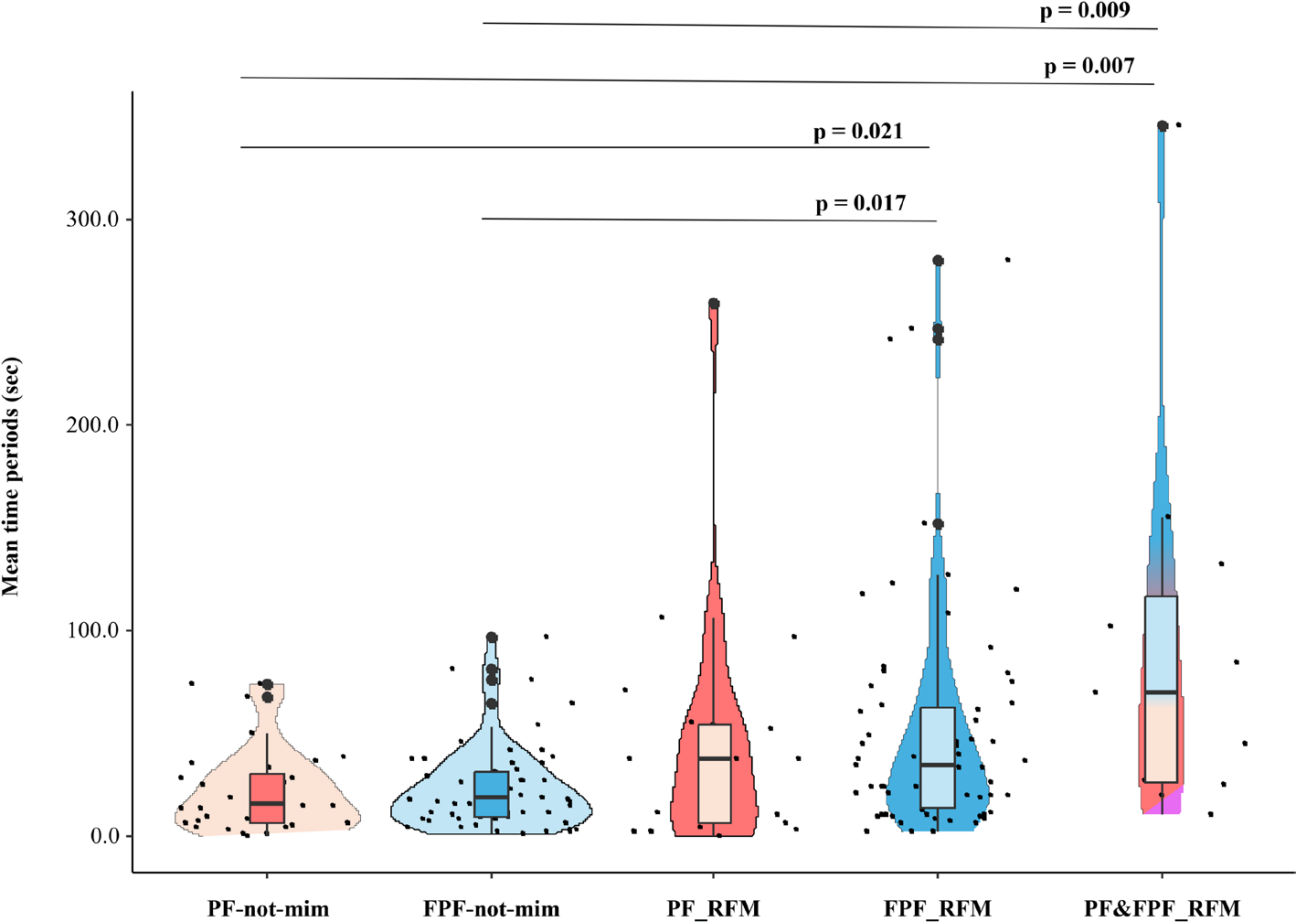
Violin plot with included boxplots representing the time period (in seconds) between the occurrence of the first playful expression or RFM event and the end of the play session across five different conditions: (i) players performed at least two not‐mimicked PFs only (*PF‐not‐mim*); (ii) players performed at least two not‐mimicked FPFs only (*FPF‐not‐mim*); (iii) at least one event of RFM of PF (but not FPF) occurred (*PF_RFM*); (iv) at least one event of RFM of FPF (but not PF) occurred (*FPF_RFM*), and (v) at least one event of both RFM of PF and RFM of FPF occurred in the same play session (*PF&FPF_RFM*). The shape of the violin represents the density estimate of the variable considered. Dots represent the individual data points (*N*_dyads_PF‐not‐mim_ = 36, *N*_dyads_FPF‐not‐mim_ = 56, *N*_dyads_PF_RFM_ = 20, *N*_dyads_FPF_RFM_ = 67, *N*_dyads_PF&FPF_RFM_ = 11). The rectangle of the small boxplot shows the ends of the first and third quartiles and the central line shows the median value. Only probabilities of significant results are representing in the graph.

*Prediction 2b*. The time period (in seconds) between the occurrence of the first playful expression (PF/FPF) or RFM event and the end of the play session significantly differed across the five conditions considered (Kruskal–Wallis test—Monte Carlo randomization *N*_time_periods_ = 186, *H* = 21.711, df = 4, *p* < 0.001; Figure 5). The *post hoc* test showed the following results: *PF‐not‐mim* versus *FPF‐not‐mim* (Bonferroni—Dunn post hoc test *Q* = −4.953, *p* = 1.000), *PF‐not‐mim* versus *PF_RFM* (*Q* = −21.466, *p* = 1.000), *PF‐not‐mim* versus *FPF_RFM* (*Q* = −35.570, *p* = 0.021), *PF‐not‐mim* versus *PF&FPF_RFM* (*Q* = −63.891, *p* = 0.007), *FPF‐not‐mim* versus *PF_RFM* (*Q* = −16.513, *p* = 1.000), *FPF‐not‐mim* versus *FPF_RFM* (*Q* = −30.617, *p* = 0.017), *FPF‐not‐mim* versus *PF&FPF_RFM* (*Q* = −58.938, *p* = 0.009), *PF_RFM* versus *FPF_RFM* (*Q* = −14.104, *p* = 1.000), *PF_RFM* versus *PF&FPF_RFM* (*Q* = −42.425, *p* = 0.358), and *FPF_RFM* versus *PF&FPF_RFM* (*Q* = −28.231, *p* = 1.000). Thus, the time periods from the occurrence of the first RFM event involving either FPF or both PF‐FPF to the end of the play session were longer compared to the time periods following the first non‐mimicked PF or FPF.

We also found that RFM events of either PF or FPF occurred more frequently before rather than after the halfway point of duration (in seconds) of each session (Binomial test *N*_RFMevents_ = 187, *p* < 0.001).

### 3.4 Prediction 3

**FIGURE 6:**
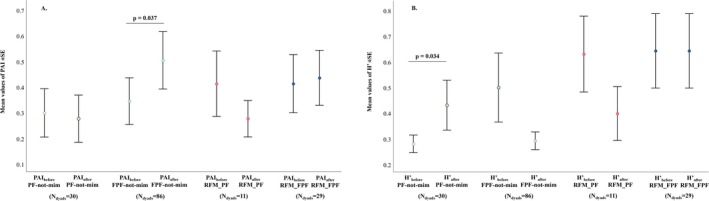
Error bars representing (A) the mean values of the Play Asymmetry Index (PAI) calculated before and after the performance of a not mimicked PF (*PF‐not‐mim*), not mimicked FPF (*FPF‐not‐mim*) and an event of PF mimicry (*PF_RFM*) and FPF mimicry (*FPF_RFM*), and (B) the mean values of the Shannon Index (*H*′) calculated before and after the performance of a not mimicked PF (*PF‐not‐mim*), not mimicked FPF (*FPF‐not‐mim*) and an event of PF mimicry (*PF_RFM*) and FPF mimicry (*FPF_RFM*). The length of bars indicates how spread the data are around the mean value (colored dots). Only probabilities of significant results are representing in the graphs. The sample size of each condition is indicated on the graph.

*Prediction 3a*. The values of PAI did not significantly differ before and after the emission of a *PF‐not‐mim* (Wilcoxon test—Monte Carlo randomization *N*_events_ = 30, *T* = 53, ties = 15, *p* = 0.685; mean PAI values ± SE: before 0.333 ± 0.060; after 0.308 ± 0.050), an event of *PF_RFM* (*N*_events_ = 11, *T* = 11.5, ties = 3, *p* = 0.449; before 0.409 ± 0.127; after 0.273 ± 0.071) and an event of *FPF_RFM* (*N*_events_ = 29, *T* = 88.5, ties = 10, *p* = 0.780; before 0.362 ± 0.060; after 0.362 ± 0.055). On the contrary, we found that PAI values were higher after than before the emission of a *FPF‐not‐mim* (*N*_events_ = 86, *T* = 847, ties = 18, *p* = 0.037; before 0.352 ± 0.037; after 0.474 ± 0.038; Figure 6A). Hence, only the occurrence of non‐mimicked FPF was followed by an increase in the asymmetry of successive play pattern exchange.

We compared all PAI values before (Kruskall–Wallis test, Monte Carlo randomization *N*_events_PF_not_mimicked_ = 30, *N*_events_FPF_not_mimicked_ = 86, *N*_events_PF_RFM_ = 11, *N*_events_FPF_RFM_ = 29, *H* = 0.277, df = 3, *p* = 0.964) and after (Kruskall–Wallis test, Monte Carlo randomization *N*_events_PF_not_mimicked_ = 30, *N*_events_FPF_not_mimicked_ = 86, *N*_events_PF_RFM_ = 11, *N*_events_FPF_RFM_ = 29, *H* = 7.497, df = 3, *p* = 0.060) a *PF‐not‐mim*, *FPF‐not‐mim*, *PF_RFM*, and *FPF_RFM*, and we did not obtain any statistical difference in both cases.

*Prediction 3b*. We found no significant difference in the Shannon Index (*H*′) values before and after the emission of a *FPF‐not‐mim* (Wilcoxon test—Monte Carlo randomization *N*_events_ = 86, *T* = 918.5, ties = 25, *p* = 0.851; mean *H*′ values ± SE: before 0.600 ± 0.054; after 0.610 ± 0.053), an event of *PF_RFM* (*N*_events_ = 11, *T* = 10.5, ties = 3, *p* = 0.345; before 0.626 ± 0.150; after 0.400 ± 0.105) and an event of *FPF_RFM* (*N*_events_ = 29, *T* = 92.5, ties = 10, *p* = 0.937; before 0.663 ± 0.100; after 0.700 ± 0.100). On the contrary, values of *H*′ were significantly higher after than before the emission of a *PF‐not‐mim* (*N*_events_ = 30, *T* = 49.0, ties = 10, *p* = 0.034; before 0.530 ± 0.100; after 0.815 ± 0.100; Figure 6B). Hence, only the occurrence of non‐mimicked PF was followed by increased variability of successive play patterns.

We compared all *H*′ values before (Kruskall–Wallis test, Monte Carlo randomization *N*_events_PF_not_mimicked_ = 30, *N*_events_FPF_not_mimicked_ = 86, *N*_events_PF_RFM_ = 11, *N*_events_FPF_RFM_ = 29, *H* = 3.201, df = 3, *p* = 0.367) and after (Kruskall–Wallis test, Monte Carlo randomization *N*_events_PF_not_mimicked_ = 30, *N*_events_FPF_not_mimicked_ = 86, *N*_events_PF_RFM_ = 11, *N*_events_FPF_RFM_ = 29, *H* = 6.902, df = 3, *p* = 0.072) a *PF‐not‐mim*, *FPF‐not‐mim*, *PF_RFM*, and *FPF_RFM*, and we did not obtain any statistical difference in both cases.

The analysis on both PAI and *H*′ included 100 play sessions.

## 4 Discussion

According to the *Complexity and Continuity Hypothesis* (Davila‐Ross and Dezecache 2021), in humans, laughter and smiles of positive affect must have evolved within the context of play in ancestral species, and these expressions have gone through a main period of evolutionary change via different phylogenetic trails in the past 10–16 million years to become effective, pervasive, and flexible patterns used during social interactions in Hominidae (Davila‐Ross and Zimmermann 2009; Davila‐Ross et al. 2008, 2015; Waller et al. 2015).

Waller et al. (2020) argued that to consider facial expressions as homologous across species (including humans) it is needed to demonstrate “a stereotyped and recognisable form…, similarity of multiple elements, homology of underlying facial musculature and presence in a large number of related species” (9).

In the current study, we showed that in lowland gorillas PF and FPF seem to maintain morphological similarities (“homology of underlying facial musculature”) with PF and FPF displayed by the other great apes (“presence in a large number of related species”; *Prediction 1a* supported; see Video S1A,B and Table S2) to the point that tools designed for the detection of the activation of AUs in humans and chimpanzees also work with minimal error for gorillas (GorillaFACS has been very recently implemented by Correia‐Caeiro et al. 2025, and it was not yet available at the time of our study).

In our study of gorillas, the performance of PF involved most of the AUs (Table S2) described for chimpanzee PF, in which the two best configurations included AU12 + AU25 + AU26 and AU12 + AU25 + AU27, with AU26 and AU27 being mutually exclusive (Parr et al. 2007). Also, in orangutans, AU26 and AU27 are involved in the PF performance, with AU27 being more activated than AU26 during higher‐intensity play sessions (Waller et al. 2015). On the other hand, in our lowland gorillas, the performance of FPF—more than that of PF—involved the activation of AUs (Table S2) responsible for upper lip raising (AU10) and mouth stretching (AU27), similar to the wide playful facial expressions observed in chimpanzees (Vick et al. 2007). In humans, laugh face and smiles (possible homologues of PF and FPF) include facial muscle movements that are similar to those of the other great apes, such as the activation of *zygomaticus mayor* (AU12) and *orbicularis oculi* (AU06) causing cheek raising and eye wrinkling (Ruch and Ekman 2001; Drack et al. 2009; Masai et al. 2022).

Our findings showed that in gorillas the PF and FPF did not completely overlap from the morphological point of view (*Prediction 1b* supported; see Table S2). As expected, conversely to PF, the muscle units associated with the exposure of higher teeth were always involved in the performance of FPF. Although occurring at low frequencies, PF and FPF may be present independently from one another during playful interactions (3.1% and 5.5% of play sessions with only PF and FPF performed, respectively) and, for the first time, we demonstrated that either PF or FPF can be rapidly replicated by players (RFM; *Prediction 2a* supported; Figure 4). Although the levels of FPF_RFM tended to be higher than those of PF_RFM, statistical significance was not achieved. Further data are needed to definitively assess any potential differences in replication levels between PF and FPF. To our knowledge, the presence of RFM in non‐human primates has been found so far by conflating PF and FPF (e.g., orang‐utan, Davila‐Ross et al. 2008; lowland gorillas and chimpanzees, Palagi, Norscia, et al. 2019; Bresciani et al. 2021; bonobos, Bertini et al. 2021). In gorillas, the RFM of both PF and FPF may serve in fostering social bonding between individuals during the positive (and safe) context of play (Bresciani et al. 2021). Also in humans, only smiles and laughter that are perceived as positive and appropriate signals may increase affiliation between individuals and this increase may be expressed via facial mimicry (Kastendieck et al. 2021). The sharing of laughter communicates a shared understanding of the context (Martin and Ford 2018).

In our gorillas, we found that the time periods (measured in sec) from the occurrence of the first RFM event involving either FPF or both PF‐FPF to the end of the play session were longer compared to the time periods following the first non‐mimicked PF or FPF (*Prediction 2b* partially supported; Figure 5). Thus, the presence of facial replication, rather than just non‐mimicked facial expressions, may contribute to prolonging the play session. Additionally, most RFM events occurred near the start of the session, suggesting they did not mark the end of the playful interaction. RFM may promote action synchronization and playful mood sharing between players (especially size‐matched players; Bresciani et al. 2021) thus favoring the maintenance of the interaction. In humans, prolonged social interactions can be associated with laughter contagion that acts as social glue (Provine 1992; Dunbar 2022). However, in our gorilla groups, we found that PF_RFM may prolong the play session mainly when it occurs alongside FPF_RFM, resulting in an amplified effect. We can hypothesize that the replication of a more noticeable signal, like FPF, may more clearly communicate the intent to continue play, thus minimizing the chances of misinterpretation and preventing play from escalating into conflict (Palagi et al. 2007; Waller and Cherry 2012).

We demonstrated that FPF—but not PF or RFM of either PF or FPF—was immediately followed by an increase in the level of play asymmetry within the session (*Prediction 3a* supported; Figure 4A). All playful sessions we analyzed involved physical contact between players (play‐fighting sessions) and the levels of FPFs per session were higher compared to those of PFs (Figure 3A), even if both signals were maintained for a comparable amount of time (Figure 3B). The frequent use of more evident signals (FPF) may better communicate the benign intent by the agent. Consequently, a clear statement of purpose may permit the subjects to turn play into a more competitive and cognitive demanding interaction, which may enhance the self‐ and social‐assessment function of play behavior (Fagen 1981; Paquette 1994; Thompson 1998). According to the *Polyvagal Theory*, play can be viewed as a neural exercise as it requires the capacity to swing between a fight/flight competitive response and a cooperative social interaction (Porges 2011). In this light, FPF can promote this transition between cooperation/competition within the session in a rapid way by maintaining the “non serious” context of play. Similarly, in preschool children exaggerated laughter (more evident signals) is most often associated with mock aggression, which is a risky form of playful interaction (Sarra and Otta 2001). Thus, from an evolutionary point of view, we may suppose that the use of more evident signals was maintained when it is needed to elicit correct behavioral responses by partners and when the sharing of context (e.g., high intensity play interactions) may be crucial for limiting the risk of misunderstanding (“similarity of multiple elements”).

Based on the *Social Brain Hypothesis* (Dunbar 2022; Shultz and Dunbar 2012) communication serves as social glue between group members. FPF in lowland gorillas may have positive social effects by improving player social assessment, such as creating and enhancing bonds between individuals (Bresciani et al. 2021). It has been recently demonstrated that in humans, more facially expressive people were more agreeable and likable to their social partners, thus suggesting that facial expressions may favor the formation and maintenance of inter‐individual social relationships (Kavanagh et al. 2022). We can suppose that this social effect of facial communication was a result of an evolutionary continuity from non‐human hominids to modern humans.

We also demonstrated that PF—but not FPF and RFM of either PF or FPF—was followed by an increase in play session variability (*Prediction 3b* supported). In this view, PF may promote in the short term the use by players of more different types of playful patterns which account for more variable—and consequently more physically and cognitively demanding—playful sessions. The increased use of different types of playful patterns within a session may facilitate the training for the unexpected (Spinka et al. 2001), as players cannot anticipate their partner's behavior but have to be able to respond appropriately to unexpected play patterns.

### 4.1 Constraints on Generality (Simons et al. 2017) and Conclusion

Although our study focused only on 21 captive lowland gorillas and caution is needed when generalizing these findings, this may be the first attempt to analyze PF and FPF and their rapid replication separately. We believe that our results may represent a starting point to encourage much more studies on potential differences between the two variants of playful facial expressions. Despite only 17 PFs and 15 FPFs being assessed by using chimpFACS and OpenFace tools (GorillaFACS was not yet available during the current study; Correia‐Caeiro et al. 2025), the consistent activation of certain AUs and the similarities with findings from other primate species (Parr et al. 2007; Vick et al. 2007; Waller et al. 2015; Masai et al. 2022) support the hypothesis that the morphology of PF and FPF has been conserved across primates. However, PF and FPF do not completely overlap morphologically and seem to have distinct functions. Specifically, PF may enhance play variability, while FPF may increase competition during playful sessions by improving play asymmetry. Thus, we hypothesize that in gorillas, PF and FPF can be preserved as distinct expressions because they likely serve different adaptive purposes. This is further supported by the observation that their RFM appears to function differently. While FPF_RFM independently contributes to prolonging play sessions compared to non‐mimicked PF or FPF, PF_RFM may require the amplified effect of FPF_RFM to achieve the same outcome. Summing, our findings may lay the groundwork for further studies to confirm or uncover new functional and/or morphological differences between playful facial expressions in both human and non‐human primates, offering a deeper understanding of the adaptive significance of maintaining two facial expressions of varying intensity throughout Hominidae evolution.

## Author Contributions

**Giada Cordoni:** conceptualization (lead), data curation (lead), formal analysis (lead), investigation (lead), methodology (lead), supervision (lead), writing – original draft (lead), writing – review and editing (lead). **Martina Brescini:** data curation (supporting), formal analysis (supporting), software (lead), writing – review and editing (supporting). **Luca Pirarba:** data curation (supporting), formal analysis (supporting). **Florinda Giaretto:** data curation (supporting), formal analysis (supporting). **Ivan Norscia:** conceptualization (lead), funding acquisition (lead), investigation (lead), methodology (lead), supervision (lead), writing – original draft (lead), writing – review and editing (supporting).

## Conflicts of Interest

The authors declare no conflicts of interest.

## Supporting information


Data S1.

## Data Availability

The datasets supporting this article have been uploaded as part of the Supporting Information S1.
