# Supplementary material for: Functional and Morphological Differences in the Play Face and Full Play Face in Lowland Gorillas, a Hominid Species: Implications for the Evolutionary Roots of Smile and Laugh Face
Source: Am J Biol Anthropol. 2025 May 8;187(1):e70061. doi: 10.1002/ajpa.70061 (PMC12062928; doi:10.1002/ajpa.70061)
Supplement: Supplementary file 1 — Data S1. [file AJPA-187-e70061-s001.zip › R3_AJBA_play_face_full_play_face_gorillas/R3_AJBA_Play_face_full_play_face_in_gorillas_MAIN_DOCUMENT_NOT_TRACKED.docx]

**FUNCTIONAL AND MORPHOLOGICAL DIFFERENCES IN THE PLAY FACE AND FULL PLAY FACE IN LOWLAND GORILLAS, A HOMINID SPECIES: IMPLICATIONS FOR THE EVOLUTIONARY ROOTS OF SMILE AND LAUGH FACE**

Giada Cordoni^a,(*)^, Martina Brescini^a^, Luca Pirarba^a^, Florinda Giaretto^a^, Ivan Norscia^a,(*)^

^a^Department of Life Sciences and System Biology - University of Torino, Via Accademia Albertina 13, Turin (Italy)

Running title: **PLAYFUL EXPRESSIONS IN LOWLAND GORILLAS**

***Corresponding authors**:

**Giada Cordoni**: giada.cordoni@unito.it

**Ivan Norscia**: ivan.norscia@unito.it

**ABSTRACT**

**Objective**. Play Face (PF) and Full Play Face (FPF) in the great apes - homologous to human smile and laugh-face - have been considered as a single phenomenon. However, if natural selection has preserved two expressions probably their adaptive value differs.

**Materials and Methods**. We collected video-data on play interactions on two lowland gorilla groups (N=21; *Gorilla gorilla gorilla*) housed at La Vallée des Singes and the ZooParc de Beauval (France). Lacking a tool tailored for gorillas during this study, we analyzed facial action-unit activation via chimpFACS and OpenFace.

**Results.** We found that PF and FPF activated partly different action-units as it occurs for chimpanzees and humans’ PF/FPF. We detected the rapid replication (Rapid Facial Mimicry-RFM) of either PF or FPF that were associated with longer play sessions. Not-mimicked PF was linked to increased play session variability (different types of play patterns) measured via the Shannon-index whereas not-mimicked FPF was associated with increased play asymmetry (imbalance between offensive/defensive patterns) measured via the Play-Asymmetry-Index.

**Discussion**. Lowland gorillas may use PF to manage sessions that are more complex in terms of pattern types and FPF - a more salient signal - to prevent misunderstandings when the session is imbalanced. RFM of both expressions may favor the prolongation of play session by increasing player synchronization and possibly emotional sharing. Our study opens the door to further comparative studies on playful expressions in humans and other primates as a way to fine-tune possible emotional communication and delineate potential evolutionary roots of Hominidae facial communication.

**Key words**: rapid facial mimicry, play asymmetry, play variability, human smile, human laugh face

**Introduction**

*Smiling, as we shall see, graduates into laughter*

(Charles Darwin, *The Expression of the Emotions in Man and Animals*)

Facial expressions - visible facial movements associated with species-specific behavioral repertoire that have a communicative meaning to group members (Waller et al., 2020) - are not a unique trait of humans. Other mammals show similar behavioral phenomena in terms of form and function, thus indicating that facial expressions have evolved long before the appearance of modern humans (Waller & Micheletta, 2013).

In *Homo sapiens*, smile and laughter occupy a central role in social cohesion, and they have probably evolved in the social context of play (Provine, 2000; Dezecache & Dunbar, 2012; Bryant et al., 2016) where human and non-human primates show common anatomical and functional features in facial behaviors (Cordoni & Palagi, 2011; Davila-Ross & Palagi, 2022). Different variants of more or less genuine smiles and laughter exist in humans (Ambadar et al., 2009; Mascarò et al., 2021). Spontaneous smile and the visual component of laughter (hereafter, laugh face) shared similar neural activation (mainly in the bilateral supplementary motor area; Iwase et al., 2002) and basic morphological features involving the contraction of face muscles such as *zygomaticus major* (lip corner pulling back and upwards), *orbicularis oculis* (crow’s feet), and *orbicularis oris* (lip pressing; Parr et al., 2007; Caeiro et al., 2013). However, smile and laugh face do not completely overlap.

Human spontaneous smile and laugh face seem to be homologous to the relaxed open mouth expressions observed almost exclusively during play in non-human primates, that is the Play Face (PF) and the Full Play Face (FPF) respectively (van Hooff & Preuschoft, 2003; Davila-Ross & Dezecache, 2021). The PF - that is almost present in all primate species (Palagi et al., 2016a) – usually involves the exposure of lower teeth, whereas during FPF - that has a more patchy distribution than PF within primates (Palagi et al., 2016a) – also the upper teeth are usually exposed due to the *levator labii superioris* muscle contraction (Davila-Ross et al., 2015; Waller et al., 2020). In chimpanzees (*Pan troglodytes*) - as in humans - both PF and FPF involve the contraction of the *zygomaticus major* (Parr et al., 2007; Waller et al., 2015, 2020). Furthermore, during FPF, chimpanzees, like humans, can exhibit parting lips, pulling lips back and upwards, jaw dropping, upper lip raising, and sometimes cheek raising (Davila-Ross et al., 2015). It is worth noting that the activation levels of the different facial muscle action units (AU) involved in the performance of both PF and FPF can show a certain degree of variability, highlighting that the two expressions represent a gradient of intensity. For example, in sanctuary chimpanzees, Davila-Ross and colleagues (2015) found that the 19% of PFs showed the activation of muscles linked to the raising of the upper lip, while Parr and colleagues (2007) did not find such activation in the Yerkes chimpanzees. For this reason, not only the exposure of the upper teeth but also the size of the mouth opening may account for the difference between PF and FPF (Davila-Ross et al., 2015).

The automatic detection and analysis of facial action units is one of the principal means for identifying and analyzing different facial expressions. Through specific toolkits (e.g. Facial Action Coding System-FACS: Ekman & Friesen, 1978; Ekman et al., 2002; OpenFace: Baltrušaitis et al., 2018), changes in facial muscle contraction related to different expressions have been identified in humans (Gilbert et al., 2021; Jeganathan et al., 2022), chimpanzees (Vick et al., 2007), rhesus macaques (Parr et al., 2010), gibbons (Waller et al., 2012), orangutans (Caeiro et al., 2013), dogs (Waller et al., 2013), cats (Caeiro et al., 2017), and horses (Wathan et al., 2015).

According to the *Complexity and Continuity Hypothesis* (Davila-Ross & Dezecache, 2021), the strong similarities in morphology and context point toward an evolutionary continuity, with no major changes, from non-human hominids to modern humans.

During playful interactions the primary function of smile/laugh face and PF/FPF is to signal the non-aggressive behavioral intentions of the agent, although with different emotional grades (Pellis & Pellis, 1996; Gervais & Wilson, 2005; Cordoni & Palagi, 2011, 2012; Palagi et al., 2022). This is particularly important during play-fighting, a form of play where many behavioral patterns are drawn from aggressive context, and physical contact between players can be sustained for an extended period (Paquette, 1994; Palagi et al., 2016a; Pellis & Pellis, 2017; Cordoni et al., 2023). The *Power Asymmetry Hypothesis* (PAH, Preuschoft & van Hooff, 1997) assumes that primate species living in despotic societies characterized by highly asymmetric hierarchical relationships need to produce unambiguous and distinct signals to distinguish play (especially, play-fighting) from other interactions such as appeasement or affiliation. Although PAH was originally framed to explain between species variation in patterns of affiliative/appeasement/playful displays, it has been suggested that it could also be applied within species that show a large variation in dominance relationships between groupmates (Mehu & Dunbar, 2008). During playful interactions, an asymmetry between players in behavioral pattern exchange and winner position attainment can occur (Bauer & Smuts, 2008; Pellis et al., 2010; Pellis & Pellis, 2017). For example, in chimpanzees, an increase in the use of playful expressions was associated with an increase in play asymmetry with the possible aim of reducing the misunderstanding between players when play becomes more competitive (Cordoni & Palagi, 2011).

In humans, smile and laugh face may convey signals with different intensities (Redican, 1982). However, smile is not only a sign of happiness (Ekman, 2003). Depending on circumstances, smile can indicate nervousness, need to please, embarrassment, welcoming attitude, and these different meanings can be related to specific variations in smile morphological and dynamic features (Ambadar et al., 2009). On the other hand, laugh face is not exclusively considered an expression of humour or happiness. Indeed, laugh face with its auditory component can be defined as a social behavior that concurs in establishing and regulating social bonds and in reducing social tension (Scott et al., 2014; Wood & Niedenthal, 2018; Palagi et al., 2022).

In non-human primates, PF and FPF partly share similar morphology and functions (e.g. prolonging the session, avoiding escalation into aggression) and are often collapsed into a single phenomenon (Pellis & Pellis, 1996, 1997; Palagi et al., 2007; Demuru et al., 2015). However, in different primate species (and all hominid species) evolution has maintained both PF and FPF signals (Davila-Ross & Dezecache, 2021). Furthermore, during play both PF and FPF they can be found also separately (Waller & Cherry, 2012; Palagi et al., 2019b). Hence, from an adaptive standpoint, it is reasonable to hypothesize that these two signals may not have fully overlapping functions. For example, compared to PF, FPF has been found especially associated with higher intensity play in gorillas (Waller & Cherry, 2012). According to literature, FPF may be especially used: i) in more tolerant societies where it is less likely that it is mistaken for threat (in despotic groups such as those of some macaque species - e.g. rhesus macaques - or baboons, the exposure of upper teeth may cause aggression by dominants; Thierry et al., 1989; van Hooff & Preuschoft, 2003); ii) when play is rough and requires the frequent display of salient play signals to communicate the non-aggressive intent of players (Palagi et al., 2016 a,b). In this respect, in lowland gorillas, play may escalate into aggression without salient play signals (Palagi et al., 2007, 2019a; Bresciani et al., 2021). Indeed, lowland gorillas (*Gorilla gorilla gorilla*) can use more FPF than chimpanzees possibly in relation to particularly rough play sessions (Palagi et al., 2019b), although no study has clearly demonstrated this point.

Both smiles and laugh face in humans and PF/FPF in great apes can act as releasing stimuli (*sensu* Tinbergen, 1952) and elicit the same facial expression in other interacting subject (see for review Palagi et al., 2020). When the replication of the same facial expression occurs within 1s from the stimulus emission, the phenomenon is known as Rapid Facial Mimicry (RFM; Dimberg & Thunberg, 1998; Sestito et al., 2013). The facial replication is involuntary and automatic (as suggested by the response speed < 1 sec) and occurs significantly more when the first stimulus (i.e. expression) is perceived by the potential receiver compared to when it is not perceived. Yet the difference between yes-perception and no-perception (of the first stimulus) conditions indicates that the phenomenon is mimicry and not simple synchronization (Palagi et al., 2020). RFM does not invoke advanced cognitive processes but rather basic automatic and involuntary processes by finding its roots in the automatic coupling of perception and action within the brain's sensorimotor areas, as foreseen by the Perception Action Model possibly involving the mirror neuron system (Gallese et al., 1996; Ferrari et al., 2003; de Waal & Preston, 2017).

RFM is present during play in both human and non-human animals showing open mouth displays (see review Palagi et al., 2020). In many primate and non-primate species the occurrence of RFM - compared to the simple presence of non-replicated play face - is associated with a longer duration of playful interaction thus a higher success of it (Tonkean macaques, Scopa & Palagi, 2016; geladas, Mancini et al., 2013; lowland gorillas, Bresciani et al., 2021; chimpanzees, Palagi et al., 2019b; orangutans, Davila-Ross et al., 2008; dogs, Palagi et al., 2015; meerkats, Palagi et al., 2019a).

In lowland gorillas - although with exceptions (Cordoni et al., 2022) - play is scarcely retained in adulthood (Masi et al., 2009; Cordoni et al., 2018). Gorillas show both PF and FPF (Palagi et al., 2007; Bresciani et al., 2021) and the occurrence of RFM - only verified by conflating PF and FPF - has been demonstrated during their social play sessions (Palagi et al., 2019b; Bresciani et al., 2021). Furthermore, gorillas have a great number of facial muscles like the other Hominoidea (e.g. zygomaticus major, orbicularis oculi, levator labii superioris; Diogo et al., 2009, 2010). Hence, gorillas are particularly suitable to investigate the evolutionary basis of the differences between smile and laugh face. To this purpose, we investigated in lowland gorillas the differences in terms of morphology (i.e. facial muscle action unit activation) and function between PF and FPF and their rapid replication (RFM). Based on the previous framework, we formulated the following predictions.

*Prediction 1*

If PF and FPF are basic expressions that have been preserved in the course of the evolution of primate facial expressions (Waller et al., 2020), we expect that - by applying the tool used for the expression analysis in other hominids (humans-OpenFace, chimpanzees-ChimpFACS) - we would detect with minimal error the activation of the same facial muscle units that activates in human and chimpanzee PF and FPF (*Prediction 1a*). Moreover, as PF and FPF should not possess the same exact morphology, we expect that the facial unites that activate during the two expressions are not fully overlapping (*Prediction 1b*).

*Prediction 2*

As RFM is present in gorillas (Palagi et al., 2019b; Bresciani et al., 2022) and PF and FPF can occur separately during play, we expected to find RFM of either PF or FPF (*Prediction 2a*). Furthermore, we expect that the duration of the playful session may be greater in the presence of RFM of either PF or FPF than in the presence of unreplicated facial signals or no signal (*Prediction 2b*).

*Prediction 3*

In gorillas, play is highly competitive and asymmetric especially between juvenile/adolescent males (Palagi et al., 2007; Cordoni et al., 2018). In this light, we expected that FPF - more of a salient signal than PF (Palagi & Mancini, 2011; Palagi et al., 2016a) - may be associated with high levels of play asymmetry to convey a clearer statement of positive mood by the agent (*Prediction 3a*). Furthermore, we also expected that in case of more symmetric interaction a less evident signal such as PF rather than FPF may be associated with an increased play variability (i.e. different types of playful behavioral patterns performed within a session; *Prediction 3b*).

**Materials and Methods**

**Ethical statement**

The current study was purely observational and non-manipulative, thus approval was not required by the authors' institutional animal care committees.

Study groups

The study was carried out on two family groups of lowland gorillas (Gorilla gorilla gorilla) housed at La Vallee des Singes (Romagne, France; hereafter, VDS) and the ZooParc de Beauval (Saint Aignan sur Cher, France; hereafter, BEA).

The VDS group was composed by 10 individuals (mean age ±SE: VDS 19.2 ±5.10 Table 1). Two adolescent males (Mawete and Djomo) were castrated. Gorillas were fed outdoors with fruit, vegetables, seeds, leaves, and trunks five times per day during spring/summer and twice per day starting from September.

The BEA group comprised 11 individuals (mean age ±SE: 16.82 ±4.52; Table 1). Two immature males (Mapenzi and Yamba) were castrated. The animals received food (fruit, vegetables, seeds, leaves, and trunks) six times per day.

VDS and BEA groups were managed in similar enclosures composing by both an indoor (VDS 150 m2, BEA 200 m2) and outdoor (a wooded island surrounded by a water canal; VDS 3400 m2, BEA 2000 m2) facilities. The enclosures were enriched with trees, lianas, trunks, straw and platforms. During the day, gorillas can move freely between the indoor and outdoor facilities and can socially interact. In both groups, silverbacks were the fathers of immature subjects, and all adult females were treated with oral contraceptive.

**Data collection**

We collected video data on both colonies during the following periods: August-October 2020/March-June 2021/March-June 2022 for VDS and June-October 2021 for BEA. For video recording, was used a full HD camera (Panasonic HDC-SD9).

The observers (L.P., F.G.) were trained by G.C. in animal recognition and the application of methodological procedures (i.e., scan and all-occurrences sampling methods; see below). Additionally, before starting the video analysis independently, the observers received training from G.C. in identifying behavioral patterns (see Table 2) and distinguishing between play face (PF) and full play face (FPF). 10% of the recorded play sessions (approximately 100 sessions) were analyzed - either in slow motion or frame-by-frame using the freeware Avidemux 2.7.8 - by both observers simultaneously and separately. Their agreement in identifying behavioral patterns and distinguishing between PF and FPF was assessed using Cohen’s Kappa, which indicates the proportion of agreement beyond what would be expected by chance. Interobserver reliability between the video coders was calculated using the R function ‘cohen.kappa’ and the ‘irr’ and ‘psych’ libraries (R version 3.5.3). Training was concluded when the interobserver reliability reached a Cohen’s Kappa values of 0.80 for PF and FPF and from 0.60 to 0.94 for playful patterns recorded in this study (see Table 2).

By scan animal sampling (Altmann, 1974) we collected at 10-min intervals the overall group daily activity by recording both solitary (moving, resting, foraging, feeding) and social (play, grooming, body contact, aggression, proximity) behaviors. Using this method, we collected a total of 254 hours of observations. Specifically, a total of 60 out of the 254 hours were spent by animals playing (60 hours of play corresponding to 360 scans). We also employed all occurrences sampling method (Altmann, 1974) for gathering data on social playful interactions: i) players' identities, ii) playful patterns in sequential order (Table 2), iii) playful expressions (PF and FPF) and their durations, and iv) play session duration. We created Excel sheets in which, for each playful session, we reported all identified play patterns (including PF and FPF) in their sequential order of occurrence as determined by video analysis. It is important to note that scans were taken while all occurrences sampling was going (a total of 254 hours of all occurrences). We analyzed a total of 1026 play-fighting sessions (the total number of play sessions in which each gorilla was involved in is reported in Table 1). All sessions involved physical contact between players and, therefore, did not differ in their intensity levels and play category. We recorded a total of 317 PFs and 831 FPFs (the total number of PFs and FPFs performed by each gorilla during their playful interactions is reported in Table 1).

*Facial unit identification - FACS and OpenFace systems*

During the current study, in absence of a tool specifically designed to detect the activation of facial muscle action units in gorillas (GorillaFACS has been very recently implemented by Correia-Caeiro et al., 2025), we employed both FACS adapted for chimpanzees (ChimpFACS; Vick et al., 2007) and OpenFace 2.0 (Ambadar et al., 2009; Baltrušaitis et al., 2018) for humans to identify which specific facial muscle action units (AU) were activated during the performance of PF and FPF by gorillas. By using FACS it is possible to compare facial behaviors independently of face morphology variability across individuals (e.g. bone structure, fatty deposit; Waller et al., 2007, 2008). FACS identifies the contraction (binary: 0=no contraction, 1=yes contraction) of 33 facial AUs and often identifies the contraction of a group of muscles instead of a single muscle.

OpenFace 2.0 can recognize facial expressions through detecting the facial AU activation (Amos et al., 2016). It can also estimate the intensity of 17 AUs (1, 2, 4, 5, 6, 7, 9, 10, 12, 14, 15, 17, 20, 23, 25, 26, and 45; Baltrušaitis et al., 2018). Since OpenFace uses a new Convolutional Neural Network based face detector and an optimized facial landmark detection algorithm, it is possible to identify AUs also when the face is non-frontal or/and in low illumination conditions. The software extracts facial characteristics by using histograms of oriented gradients and reduces dimensionality by using PCA. Subject-specific neutral expression is extracted by computing the median value of face descriptors in the video sequence, assuming that most frames contained neutral expressions. The extracted median face is subtracted from the feature descriptor, leading to a normalized feature. The normalized feature vector describes the dynamic change from the neutral expression. The AU recognition framework uses linear kernel support vector machines for AU occurrence detection and support vector regression for AU intensity estimation (Baltrušaitis et al., 2018).

We applied both FACS and OpenFace to evaluate the AU activation of PFs (for 8 selected gorillas) and FPFs (for 9 selected gorillas; see Fig. 1 and videos SOM1A and SOM1B). For each selected individual, at least one PF and one FPF were analyzed (see Table SOM2). Since FACS and OpenFace were not designed for gorillas, we selected the facial expressions (PF and FPF) that could be very clearly seen on videos and mostly frontal for a preliminary assessment. It is important to note that the main purpose of this part of the research was to verify that PF and FPF were different, not to provide definitive and fine indications of all the AUs activated, especially since that would require a GorillaFACS, which is not yet available at the time of our study (Correia-Caeiro et al., 2025). M.B. coded these expressions with both FACS (manuals for coding were used) and OpenFace and confirmed the types of play faces determined by the observers (L.P., F.G.) before the coding with software. Then, I.N. and G.C. re-coded the PFs and FPFs of the selected gorillas by FACS and OpenFace, respectively. The inter-coder reliability reached a Cohen's K value equal to 0.81 (17 PFs and 15 FPFs were assessed).

*Operational definitions*

*Play session*

A play session started when one individual directed any playful behavioral patterns (Table 2) towards a conspecific and ended when one of the players or a third uninvolved individual interrupted the interaction (Cordoni et al., 2021, 2022). The mean duration (in sec) of play session was 42.80 ±2.10 SE, with minimum value 5.0 sec and maximum value 405.0 sec. We distinguished behaviors in offensive (*O*-behaviors of attack towards the partners), defensive (*D*-behaviors of avoidance/protection towards the attack by the partner) and neutral (*N*-neither offensive nor defensive behaviors; see Table 2; Cordoni et al., 2021, 2022, 2023).

*PF and FPF duration*

The duration of all PFs and FPFs observed in the collected videos was calculated by a frame-by-frame video analysis starting from the first frame in which the gorilla's lips were separated until the first frame in which the lips were closed again (Bertini et al., 2021; Bresciani et al., 2022). The inter-observer reliability for the duration of the two types of playful expressions was assessed using the mean-duration-per-occurrence inter-observer-agreement (IOA) for high number of timings (Reed & Azulay, 2010). We selected about 10% of PFs (durations of 40 PFs assessed) and FPFs (durations of 90 FPFs assessed) recorded on videos for calculating the IOA. The duration of each PF/FPF (i.e. duration measurement trial) was evaluated by the two observers separately. IOA was determined for each duration measurement trial by dividing the smaller duration on the higher duration reported by the two observers. For example, if the duration of a PF was 15 sec for one observer and 20 sec for the other observer, the IOA for this duration measurement trial was 15/20=0.75. IOA values of all duration measurement trials were summed and divided for the total number of trials; the result was transformed in %. The IOA for PF and FPF durations was equal to 93%.

*The evaluation of the presence of the Rapid Facial Mimicry (RFM).*

To demonstrate the presence of RFM in the groups under study, each time a player (hereafter, the trigger) emitted the first PF/FPF (hereafter, first stimulus), we evaluated the presence or absence of a PF/FPF emitted by the play partner (hereafter, the potential responder) within 1 sec after the emission of the first stimulus under two perception conditions (Fig. 2): the first stimulus was perceived by the potential responder (i.e. the stimulus fell within the visual field of the responder; *yes-perception* condition) and the first stimulus was not perceived by the potential responder (i.e. the stimulus did not fall within the visual field of the responder; *no-perception* condition). All doubtful cases (e.g. when observers did not clearly see the faces of the players from the video, or the head positions of the players were not clear) were discarded from the analysis. The agreement between the two observers in determining both the *yes-perception* and *no-perception* condition was measured across 300 play sessions (approximately 30% of the total play sessions analyzed), including 15 gorillas for *yes-/no-perception* of PF and 12 gorillas for *yes-/no-perception* of FPF. Cohen’s k reached a value of 0.82 for *yes-/no-perception* of PF and 0.83 for *yes-/no-perception* of FPF. We complemented this analysis with another that considered how many PFs/FPFs emitted by the trigger were perceived *vs* not perceived to the potential responder and how many of these PFs/FPFs were replicated or not replicated by the potential responder within 1 second after their emission. Based on the definition of RFM (see Introduction), all these analyses included only congruent responses by the potential responder to the first stimulus: a PF in response to a PF and a FPF in response to a FPF (exact facial matching).

We then compared the individual levels of PF_RFM with individual levels of FPF_RFM by dividing the number of PFs/FPFs replicated by the potential responder on the number of PFs/FPFs they perceived.

*Play session duration*

To evaluate possible effects of PF, FPF or RFM on play session duration, we considered five different conditions: i) players performed at least two not-mimicked PFs only (*PF-not-mim*); ii) players performed at least two not-mimicked FPFs only (*FPF-not-mim*); iii) at least one event of RFM of PF (but not FPF) occurred (*PF_RFM*); iv) at least one event of RFM of FPF (but not PF) occurred (*FPF_RFM*), and v) at least one event of both RFM of PF and RFM of FPF occurred in the same play session (*PF&FPF_RFM*). For each condition, we determined the time period (in sec) between the occurrence of the first PF, FPF or RFM event and the end of the play session (i.e. the time period remaining from the first PF/FPF/RFM). Then, we compared these time periods across the five conditions. We excluded from the analysis those play sessions in which no PF/FPF or RFM events occurred, as well as ambiguous cases where not-mimicked PF and FPF were both present in the same session. Furthermore, to determine whether RFM of either PF or FPF occurred near the start (for prolonging the playful session) or the end (for signaling the end of the playful session) of a play session, we calculated the halfway point of duration (in sec) of each session. We then evaluated whether the first RFM event occurred before (near the session start) or after (near the session end) this midpoint (a binomial variable: before the midpoint = 1; after the midpoint = 0).

*Play Asymmetry Index (PAI)*

PAI was calculated by considering the number of offensive and defensive behavioral patterns (see Table 2) exchanged between players on the total number of patterns (offensive + defensive + neutral) composing the session (Cordoni et al., 2018, 2023):

$$PAI=\frac{\left( {offensive}_{A\to B}+{defensive}_{B\to A} \right)- ({offensive}_{B\to A}+ {defensive}_{A\to B})}{(offensive+defensive+neutral)}$$

In the formula, A and B represent the players and the arrow ($\to)$ who directed behaviors to whom. PAI was calculated before and after a PF/FPF or RFM event (see sequential analysis description below) and ranged from -1 to +1. PAI values equal to or near -1 indicate a complete asymmetry in favor of player B, who performed more offensive/fewer defensive patterns or received fewer offensive/more defensive patterns from player A. PAI values equal to or near +1 indicate complete asymmetry in favor of player A. PAI values equal to or near 0 indicate complete symmetry in the exchange of offensive and defensive behaviors between players.

*Shannon Index (H’)*

The H' (Shannon, 1948; Keylock, 2005) was an ecological index that it was adapted for evaluating the level of play variability in terms of different types of behavioral patterns performed by players during a session (Cordoni et al., 2023). All offensive, defensive, and neutral patterns described in Table 2 were considered for the calculation of H’. The index was calculated as follows:

H' *= - Σ [(n_i_ / N) * (ln n_i_ / N)]*

In the formula, n*_i_* represented the number of behaviors belonging to the type *i* and *N* represented the total number of behaviors composing a session. For example, for a session composed by "*play slapping - play slapping - play sheltering - play slapping - pirouetting*", n*_i_*=3 (behavioral types=*play slapping*, *play sheltering*, and *pirouetting*), N=5 (total number of behavioral patterns composing the session that is 3 *play slapping* + 1 *play sheltering* + 1 *pirouetting*). A high value of H' indicates a great behavioral pattern variability.

*Sequential analysis*

To evaluate possible variations in play asymmetry and variability within a session related to the occurrence of PF, FPF, or RFM, we conducted a sequential analysis on each play session, as detailed in the Excel sheets (see Data Collection). When a PF, FPF, or RFM (of both PF and FPF) event occurred within a session, we selected - if possible - the four play patterns before and the four play patterns after a playful expression (PF or FPF) or mimicry event (PF_RFM or FPF_RFM). Based on these patterns, we calculated the PAI and H’ values before and after a PF, FPF or an RFM event. Consequently, for each PF, FPF, or RFM event within a session, we obtained PAI_before_, PAI_after_, H’_before_, and H’_after_. We selected four patterns before and after a playful expression or RFM event to standardize the data and give the probability to each player to perform at least one offensive and one defensive pattern. Furthermore, because there may be a session duration bias (high variability in session duration), we standardized these analyses by selecting four patterns before and four patterns after. When multiple playful expressions or RFM events occurred consecutively within a session, we calculated PAI_before_, PAI_after_, H’_before_, and H’_after_ only if the two expressions or RFM events were separated by at least four play patterns (excluding the four patterns used for index calculation). Thus, we excluded from the analysis consecutive playful expressions or RFM events separated by fewer than four patterns.

*Statistical analyses*

The distribution of time periods in the five conditions (see Operational definitions) was not normal (Shapiro-Wilk test 0.643≤W≤0.862; 0.001≤p≤0.029) thus we employed the non-parametric Kruskal-Wallis test used for k-independent samples. We also applied the Monte Carlo randomization (10.000 permutations) because of the non-independence of data due to the fact that a same individual could be present in more than one dyad. In case of test significance, we applied a post-hoc test with Bonferroni correction for pairwise comparison.

The median durations of PF/FPF, AU intensity values, levels of PF_RFM and FPF_RFM, and PAI/H' values (Shapiro-Wilk test 0.612≤W≤0.800; 0.001≤p≤0.007) were compared at the individual level by employing the non-parametric Wilcoxon exact test for 2 dependent samples with Bonferroni’s correction when necessary.

We used a binomial test to evaluate whether the first RFM event within the session occurred more frequently before or after the halfway point of duration (in sec) of each session.

A 2x2 contingency chi-square test was used to compare the number of PFs/FPFs replicated or not replicated within 1 second by the potential responder under perceived and not-perceived conditions. To ensure more conservative results, Yates’ Continuity Correction was applied.

To compare the individual number of PF/FPF performed per play session (Shapiro-Wilk test 0.146≤W≤0.183; 0.066≤p≤0.200), we employed the parametric paired-t-test for dependent samples.

To demonstrate the occurrence of RFM for both PF and FPF we ran two Generalized Linear Mixed Models (GLMM). The first model (GLMM_PF_) included as target variable the presence/absence (binomial variable; 0=absence, 1=presence) of a PF performed by the potential responder within 1 sec after the emission of the first PF by the trigger. The fixed factors were the perception condition of the potential responder (binomial variable; 0=*no-perception*, 1=*yes-perception*) and the group (binomial variable; 1=BEA group, 2=VDS group). The dyad IDs (trigger-potential responder) were entered as random factors.

The second model (GLMM_FPF_) included as target variable the presence/absence (binomial variable; 0=absence, 1=presence) of a FPF performed by the potential responder within 1 sec after the emission of the first FPF by the trigger. The fixed factors were the perception condition of the potential responder (binomial variable; 0=*no-perception*, 1=*yes-perception*) and the group (binomial variable; 1=BEA group, 2=VDS group). The dyad IDs (trigger-potential responder) were entered as random factors.

**Results**

*Preliminary analyses*

We carried out this analysis at the individual level by comparing the number of PFs/FPFs performed per play session (i.e. total number of PFs/FPFs performed by an individual divided by total number of play sessions in which this individual was involved). We found that levels of FPFs were higher than levels of PFs (paired t-test N_individuals_=21, gl=20, t=-2.608, p=0.017; mean value of PFs/FPFs per session ±SE: PF 0.26 ±0.05; FPF 0.51 ±0.11; Fig. A.3).

For each individual, we also calculated the medians of both PF and FPF durations. The median durations did not significantly differ between PF and FPF (Wilcoxon exact test N_individuals_=16, T=56.0, ties=1, p=0.835; Fig. B.3). The sample size is 16 because this analysis included only individuals for whom both PF and FPF were recorded (see Table 1).

The percentages of play sessions during which only one type of playful expression was performed were 3.1% and 5.5% for PF and FPF, respectively.

*Prediction 1*

Through the use of chimpFACS, during the performance of PFs we detected the activation of the AUs reported in the Table SOM2 and Figure SOM3. We obtained 14 different configurations for PF and - even though the sample size is limited (N=8 gorillas) - we found that four AUs were always or almost always present in the configurations detected: AU12 (*zygomaticus major* - lip corner puller), AU16 (*depressor labii inferioris* – lower lip depressor), AU25 (*depressor labii*, *orbicularis oris* - lips part), and AU26 (non-mimetic muscle - jaw drop). We also reported in the Table SOM2 and Figure SOM4 the activation of the AUs during the performance of FPFs (N=9 gorillas). We obtained six different configurations for FPF and eight AUs were always or almost always present in the configurations detected: AU06 (*orbicularis oculi*, *pars orbitalis* - cheek raiser), AU09 (*levator labii superioris alaquae nasi* - nose wrinkler), AU10 (*levator labii superioris* - upper lip raiser), AU12, AU16, AU25, AU26, and AU27 (non-mimetic muscle – mouth stretch). AU10 was activated only twice and AU27 only once during the performance of PFs thus suggesting that PF and FPF may differ for a certain extent in upper lip raising and mouth stretch.

By OpenFace 2.0, we obtained the intensity values of the AUs involved in the performance of both PF and FPF (see raw data provided as supporting material). OpenFace does not estimate the intensity value of both AU16 and AU27; for this reason, the following analysis did not include these two AUs. For each AU, we compared the intensity values between PF and FPF at the individual level and we obtained the following results (Bonferroni's correction α=0.005): AU1 - inner brow raiser (Wilcoxon exact test N_individuals_=8, T=4, ties=2, p=0.219), AU2 outer brow raiser (N_individuals_=8, T=0, ties=0, p=1.000), AU6 (N_individuals_=8, T=10.5, ties=0, p=0.328), AU9 (N_individuals_=8, T=5, ties=3, p=0.498), AU10 (N_individuals_=8, T=0, ties=0, p=0.008), AU12 (N_individuals_=8, T=4.5, ties=0, p=0.063), AU17 - chin raiser (N_individuals_=8, T=3, ties=0, p=0.039), AU 25 (N_individuals_=8, T=10, ties=0, p=0.313), AU 26 (N_individuals_=8, T=11, ties=0, p=0.383), and AU45 - blink (N_individuals_=8, T=13.5, ties=1, p=0.984). Although no comparison reached statistical significance, the probability of AU10 was the closest to significance when considering the Bonferroni’s correction. We can infer that AU10 intensity tended to be higher during FPF than PF performance.

Hence, the results of both chimpFACS and human OpenFace suggested that PF and FPF may differ principally in the activation of AU10 (higher lip raising) and AU27 (mouth stretching) that are mainly responsible for the exposure of upper teeth.

*Prediction 2*

*Prediction 2a*. We ran GLMM_PF_ to verify the occurrence of PF_Rapid Facial Mimicry (PF_RFM). The full model (including the fixed factors) and the null model (only including the random factors) significantly differed (likelihood ratio test: χ^2^=8.281, df=2, p=0.016). Because the predictor had a significant effect on the target variable, we applied the drop1 procedure. We found that the perception condition significantly affected the target variable: the probability of a PF emission by the potential responder was higher when they visually perceived the PF emitted by the trigger (*yes-perception* condition) compared to when they did not visually perceive (*no-perception* condition) the first stimulus (Table 3). The 2x2 contingency chi-square test revealed that PFs were significantly more replicated when they were perceived by the potential responder compared to the other conditions (χ^2^ = 5.080, df=1, p=0.024; Fig. A.4).

GLMM_FPF_ was carried out to verify the occurrence of FPF_Rapid Facial Mimicry (FPF_RFM). The full model and the null model significantly differed (likelihood ratio test: χ^2^=50.239, df=2, p<0.001). We found that the perception condition significantly affected the target variable: the probability of a FPF emission by the potential responder was higher when they visually perceived the FPF emitted by the trigger (*yes-perception* condition) compared to when they did not visually perceive (*no-perception* condition) the first stimulus (Table 3). The 2x2 contingency chi-square test showed that FPFs were significantly more replicated when they were perceived by the potential responder compared to the other conditions (χ^2^ =45.024, df=1, p<0.001; Fig. B.4). Summing, the RFM was determined for both PF and FPF separately.

The levels of FPF_RFM tended to be higher than the levels of PF_RFM (Wilcoxon exact test N=10, T=7.0, ties=1.0, p=0.074; mean level of RFM ±SE: PF_RFM 0.31 ±0.08; FPF_RFM 0.57 ±0.10). The sample size was smaller (N=10) because the analysis only included individuals who had both perceived and replicated a PF and a FPF.

*Prediction 2b*. The time period (in sec) between the occurrence of the first playful expression (PF/FPF) or RFM event and the end of the play session significantly differed across the five conditions considered (Kruskal-Wallis test - Monte-Carlo randomization N_time_periods_=186, H=21.711, df=4, p<0.001; Fig. 5). The *post-hoc* test showed the following results: *PF-not-mim* *vs* *FPF-not-mim* (Bonferroni - Dunn post-hoc test Q=-4.953, p=1.000), *PF-not-mim* *vs* *PF_RFM* (Q=-21.466, p=1.000), *PF-not-mim* *vs* *FPF_RFM* (Q=-35.570, p=0.021), *PF-not-mim* *vs* *PF&FPF_RFM* (Q=-63.891, p=0.007), *FPF-not-mim* *vs* *PF_RFM* (Q=-16.513, p=1.000), *FPF-not-mim* *vs* *FPF_RFM* (Q=-30.617, p=0.017), *FPF-not-mim* *vs* *PF&FPF_RFM* (Q=-58.938, p=0.009), *PF_RFM vs* *FPF_RFM* (Q=-14.104, p=1.000), *PF_RFM vs* *PF&FPF_RFM* (Q=-42.425, p=0.358), and *FPF_RFM vs* *PF&FPF_RFM* (Q=-28.231, p=1.000). Thus, the time periods from the occurrence of the first RFM event involving either FPF or both PF-FPF to the end of the play session were longer compared to the time periods following the first non-mimicked PF or FPF.

We also found that RFM events of either PF or FPF occurred more frequently before rather than after the halfway point of duration (in sec) of each session (Binomial test N_RFMevents_=187, p<0.001).

*Prediction 3*

*Prediction 3a*. The values of Play Asymmetry Index (PAI) did not significantly differ before and after the emission of a *PF-not-mim* (Wilcoxon test - Monte-Carlo randomization N_events_=30, T=53, ties=15, p=0.685; mean PAI values ±SE: before 0.333 ±0.060; after 0.308 ±0.050), an event of *PF_RFM* (N_events_ =11, T=11.5, ties=3, p=0.449; before 0.409 ±0.127; after 0.273 ±0.071) and an event of *FPF_RFM* (N_events_ =29, T=88.5, ties=10, p=0.780; before 0.362 ±0.060; after 0.362 ±0.055). On the contrary, we found that PAI values were higher after than before the emission of a *FPF-not-mim* (N_events_ =86, T=847, ties=18, p=0.037; before 0.352 ±0.037; after 0.474 ±0.038; Fig. A.6). Hence, only the occurrence of non-mimicked FPF was followed by an increase of the asymmetry of successive play pattern exchange.

We compared all PAI values before (Kruskall Wallis test, Monte Carlo randomization N_events_PF_not_mimicked_=30, N_events_FPF_not_mimicked_=86, N_events_PF_RFM_=11, N_events_FPF_RFM_=29, H=0.277, df=3, p=0.964) and after (Kruskall Wallis test, Monte Carlo randomization N_events_PF_not_mimicked_=30, N_events_FPF_not_mimicked_=86, N_events_PF_RFM_=11, N_events_FPF_RFM_=29, H=7.497, df=3, p=0.060) a *PF-not-mim*, *FPF-not-mim*, *PF_RFM*, and *FPF_RFM* and we did not obtain any statistical difference in both cases.

*Prediction 3b*. We found no significant difference in the Shannon Index (H') values before and after the emission of a *FPF-not-mim* (Wilcoxon test - Monte-Carlo randomization N_events_ =86, T=918.5, ties=25, p=0.851; mean H' values ±SE: before 0.600 ±0.054; after 0.610 ±0.053), an event of *PF_RFM* (N_events_ =11, T=10.5, ties=3, p=0.345; before 0.626 ±0.150; after 0.400 ±0.105) and an event of *FPF_RFM* (N_events_ =29, T=92.5, ties=10, p=0.937; before 0.663 ±0.100; after 0.700 ±0.100). On the contrary, values of H' were significantly higher after than before the emission of a *PF-not-mim* (N_events_ =30, T=49.0, ties=10, p=0.034; before 0.530 ±0.100; after 0.815 ±0.100; Fig. B.6). Hence, only the occurrence of non-mimicked PF was followed by increased variability of successive play patterns.

We compared all H’ values before (Kruskall Wallis test, Monte Carlo randomization N_events_PF_not_mimicked_=30, N_events_FPF_not_mimicked_=86, N_events_PF_RFM_=11, N_events_FPF_RFM_=29, H=3.201, df=3, p=0.367) and after (Kruskall Wallis test, Monte Carlo randomization N_events_PF_not_mimicked_=30, N_events_FPF_not_mimicked_=86, N_events_PF_RFM_=11, N_events_FPF_RFM_=29, H=6.902, df=3, p=0.072) a *PF-not-mim*, *FPF-not-mim*, *PF_RFM*, and *FPF_RFM* and we did not obtain any statistical difference in both cases.

The analysis on both PAI and H’ included 100 play sessions.

**Discussion**

According to the *Complexity and Continuity Hypothesis* (Davila-Ross & Dezecache, 2021), in humans laughter and smile of positive affect must have evolved within the context of play in ancestral species and these expressions have gone through a main period of evolutionary change via different phylogenetic trails in the past 10-16 million years to become effective, pervasive and flexible patterns used during social interactions in Hominidae (Davila-Ross & Zimmermann, 2009; Davila-Ross et al., 2008, 2015; Waller et al., 2015).

Waller and colleagues (2020) argued that to consider facial expressions as homologous across species (including humans) it is needed to demonstrate "*a stereotyped and recognisable form..., similarity of multiple elements, homology of underlying facial musculature and presence in a large number of related species*" (p.9).

In the current study, we showed that in lowland gorillas play face (PF) and full play face (FPF) seem to maintain morphological similarities ("*homology of underlying facial musculature"*) with PF and FPF displayed by the other great apes ("*presence in a large number of related species"; Prediction 1a* supported; see videos SOM1A and SOM1B and Table SOM2) to the point that tools designed for the detection of the activation of action units in humans and chimpanzees also work with minimal error for gorillas (GorillaFACS has been very recently implemented by Correia-Caeiro et al., 2025 and it was not yet available at the time of our study).

In our study gorillas, the performance of PF involved most of the AUs (Table SOM2) described for chimpanzee PF in which the two best configurations included AU12+AU25+AU26 and AU12+AU25+AU27, with AU26 and AU27 being mutually exclusive (Parr et al., 2007). Also in orangutans, AU26 and AU27 are involved in the play face performance with AU27 being more activated than AU26 during higher intensity play sessions (Waller et al., 2015). On the other hand, in our lowland gorillas the performance of FPF - more than that of PF - involved the activation of AUs (Table SOM2) responsible for upper lip raising (AU10) and mouth stretching (AU27), similar to the wide playful facial expressions observed in chimpanzees (Vick et al., 2007). In humans, laugh face and smiles (possible homologues of PF and FPF) include facial muscle movements that are similar to those of the other great apes such as the activation of *zygomaticus mayor* (AU12) and *orbicularis oculi* (AU06) causing cheek raising and eye wrinkling (Ruch & Ekman, 2001; Drack et al., 2009; Masai et al., 2022).

Our findings showed that in gorillas the PF and FPF did not completely overlap from the morphological point of view (*Prediction 1b* supported; see Table SOM2). As expected, conversely to PF, the muscle units associated with the exposure of higher teeth were always involved in the performance of FPF. Although occurring at low frequencies, PF and FPF may be present independently from one another during playful interactions (3.1% and 5.5% of play sessions with only PF and FPF performed, respectively) and, for the first time, we demonstrated that either PF or FPF can be rapidly replicated by players (RFM; *Prediction 2a* supported; Figure 4). Although the levels of FPF_RFM tended to be higher than those of PF_RFM, statistical significance was not achieved. Further data are needed to definitively assess any potential differences in replication levels between PF and FPF. To our knowledge, the presence of RFM in non-human primates has been found so far by conflating PF and FPF (e.g. orang-utan, Davila-Ross et al., 2008; lowland gorillas and chimpanzees, Palagi et al., 2019b; Bresciani et al., 2021; bonobos, Bertini et al., 2021). In gorillas the RFM of both PF and FPF may serve in fostering social bonding between individuals during the positive (and safe) context of play (Bresciani et al., 2021). Also in humans, only smiles and laughter that are perceived as positive and appropriate signals may increase affiliation between individuals and this increasing may be expressed via facial mimicry (Kastendieck et al., 2021). The sharing of laughter communicates a shared understanding of the context (Martin, 2018).

In our gorillas, we found that the time periods (measured in sec) from the occurrence of the first RFM event involving either FPF or both PF-FPF to the end of the play session were longer compared to the time periods following the first non-mimicked PF or FPF (*Prediction 2b* partially supported; Fig. 5). Thus, the presence of facial replication, rather than just non-mimicked facial expressions, may contribute to prolong the play session. Additionally, most RFM events occurred near the start of the session, suggesting they did not mark the end of the playful interaction. RFM may promote action synchronization and playful mood sharing between players (especially size-matched players; Bresciani et al., 2021) thus favoring the maintenance of the interaction. In humans, prolonged social interactions can be associated with laughter contagion that acts as social glue (Provine, 1992; Dunbar, 2022). However, in our gorilla groups, we found that PF_RFM may prolong the play session mainly when it occurs alongside FPF_RFM resulting in an amplified effect. We can hypothesize that the replication of a more noticeable signal, like FPF, may more clearly communicate the intent to continue play, thus minimizing the chances of misinterpretation and preventing play from escalating into conflict (Palagi et al., 2007; Waller & Cherry, 2012).

We demonstrated that FPF - but not PF or RFM of either PF or FPF - was immediately followed by an increase in the level of play asymmetry within the session (*Prediction 3a* supported; Fig. A4). All playful sessions we analyzed involved physical contact between players (play-fighting sessions) and the levels of FPFs per session were higher compared to those of PFs (Fig. A3), even if both signals were maintained for a comparable amount of time (Fig. B3). The frequent use of more evident signals (FPF) may better communicate the benign intent by the agent. Consequently, a clear statement of purpose may permit the subjects to turn play into a more competitive and cognitive demanding interaction, which may enhance the self- and social-assessment function of play behaviour (Fagen, 1981; Paquette, 1994; Thompson, 1998). According to the *Polyvagal Theory*, play can be viewed as a neural exercise as it requires the capacity to swing between a fight/flight competitive response and a cooperative social interaction (Porges, 2011). In this light, FPF can promote this transition between cooperation/competition within the session in a rapid way by maintaining the "non serious" context of play. Similarly, in preschool children exaggerated laughter (more evident signals) is most often associated with mock aggression, which is a risky form of playful interaction (Sarra & Otta, 2001). Thus, from an evolutionary point of view, we may suppose that the use of more evident signals was maintained when it is needed to elicit correct behavioral responses by partners and when the sharing of context (e.g. high intensity play interactions) may be crucial for limiting the risk of misunderstanding ("*similarity of multiple elements"*).

Based on the *Social Brain Hypothesis* (Dunbar, 2022; Shultz & Dunbar, 2012) communication serves as social glue between group members. FPF in lowland gorillas may have positive social effects by improving player social assessment such as creating and enhancing bonds between individuals (Bresciani et val., 2021). It has been recently demonstrated that in humans more facially expressive people were more agreeable and likeable by their social partners thus suggesting that facial expressions may favor the formation and maintenance of inter-individual social relationships (Kavanagh et al., 2022). We can suppose that this social effect of facial communication was a result of an evolutionary continuity from non-human hominids to modern humans.

We also demonstrated that PF - but not FPF and RFM of either PF or FPF - was followed by an increase in play session variability (*Prediction 3b* supported). In this view, PF may promote in the short term the use by players of more different types of playful patterns which account for more variable - and consequently more physically and cognitive demanding - playful sessions. The increased use of different types of playful patterns within a session may facilitate the training for the unexpected (Ŝpinka et al., 2001), as players cannot anticipate their partner's behavior but have to be able to respond appropriately to unexpected play patterns.

*Constraints on generality (Simons et al., 2017) and conclusion*

Although our study focused only on 21 captive lowland gorillas and caution is needed when generalizing these findings, this may be the first attempt to analyze PF and FPF and their rapid replication separately. We believe that our results may represent a starting point to encourage much more studies on potential differences between the two variants of playful facial expressions. Despite only 17 PFs and 15 FPFs were assessed by using chimpFACS and OpenFace tools (GorillaFACS was not yet available during the current study; Correia-Caeiro et al., 2025), the consistent activation of certain AUs and the similarities with findings from other primate species (Parr et al., 2007; Vick et al., 2007; Waller et al., 2015; Masai et al., 2022) support the hypothesis that the morphology of PF and FPF has been conserved across primates. However, PF and FPF do not completely overlap morphologically and seem to have distinct functions. Specifically, PF may enhance play variability, while FPF may increase competition during playful sessions by improving play asymmetry. Thus, we hypothesize that in gorillas, PF and FPF can be preserved as distinct expressions because they likely serve different adaptive purposes. This is further supported by the observation that their rapid facial mimicry (RFM) appears to function differently. While FPF_RFM independently contributes to prolong play sessions compared to non-mimicked PF or FPF, PF_RFM may require the amplified effect of FPF_RFM to achieve the same outcome. Summing, our findings may lay the groundwork for further studies to confirm or uncover new functional and/or morphological differences between playful facial expressions in both human and non-human primates, offering a deeper understanding of the adaptive significance of maintaining two facial expressions of varying intensity throughout Hominidae evolution.

**Acknowledgments**

Thanks are due to the staff of *La Vallée des Singes* and the *ZooParc de Beauval,* Baptiste Mulot and Jean Pascal Guéry and the gorilla keepers for allowing this study. Funding support for the conduct of this research was provided to I.N. by the Department of Life Sciences and Systems Biology (DBIOS), University of Torino, Italy (Grant No. NORI_RILO_18_01)

**Author Contribution**

**G.C., I.N.:** conceptualization, data curation, formal analysis, methodology, supervision, writing - original draft preparation, writing - review and editing. **M.B.**: data curation, video-analysis, software processing, editing. **L.P., F.G.**: video-analysis.

**Data Availability Statement**

The datasets supporting this article have been uploaded as part of the supplementary material

**Declaration of Generative AI and AI assisted technologies in the writing process**

During the preparation of this work the authors did not use any Generative AI or AI assisted technology

**Conflict of interest**

The authors have no conflict of interests

**References**

Altmann, J. (1974). Observational study of behavior: Sampling methods. *Behaviour,* 49*,* 227-267. http://dx.doi.org/10.1163/156853974X00534

Ambadar, Z., Cohn, J. F., & Reed, L. I. (2009). All smiles are not created equal: morphology and timing of smiles perceived as amused, polite, and embarrassed/ nervous. *Journal of Nonverbal Behavior*, 33, 17-34. https://doi.org/10.1007/s10919-008-0059-5

Amos, B., Ludwiczuk, B., & Satyanarayanan, M. (2016). Openface: a general-purpose face recognition library with mobile applications. CMU-CS-16-118, CMU School of Computer Science, Tech. Rep.

Baltrušaitis, T., Zadeh, A., Lim, Y.C., & Morency, LP. (2018). OpenFace 2.0: facial behavior analysis toolkit.  *IEEE International Conference on Automatic Face and Gesture Recognition*.

Bauer, E. B., & Smuts, B. B. (2007). Cooperation and competition during dyadic play in domestic dogs, *Canis familiaris*. *Animal Behaviour*, 73(3), 489-499. https://doi.org/10.1016/j.anbehav.2006.09.006.

Bertini, M., Annicchiarico, G., Bresciani, C., Cordoni, G., & Palagi, E. (2021). Playful interactions and facial mimicry in infant bonobos (*Pan paniscus*). *Ethology, Ecology & Evolution*, 34(3), 344-359. https://doi.org/10.1080/03949370.2021.1988723

Bresciani, C., Cordoni, G., & Palagi E. (2021). Playing together, laughing together: Rapid facial mimicry and social sensitivity in lowland gorillas. *Current Zoology*, 68(5), 560-569. https://doi.org/10.1093/cz/zoab092

Bryant, G. A., et al. (2016). Detecting affiliation in colaughter across 24 societies. *Proceedings of the National Academy of Sciences of the United States of America,* 113(17), 4682-4687. https://doi.org/10.1073/pnas.1524993113

Caeiro, C. C., Burrows, A. M., & Waller, B. M. (2017). Development and application of CatFACS: Are human cat adopters influenced by cat facial expressions? *Applied Animal Behaviour Science*, 189, 66-78. https://doi.org/10.1016/j.applanim.2017.01.005.

Caeiro, C. C., Waller, B. M., Zimmermann, E., Burrows, A. M., & Davila-Ross, M. (2013). OrangFACS: A Muscle-Based Facial Movement Coding System for Orangutans (Pongo spp.). *International Journal of Primatology*, 34, 115-129. https://doi.org/10.1007/s10764-012-9652-x

Cordoni, G., & Palagi, E. (2011). Ontogenetic trajectories of chimpanzee social play: similarities with humans. *PLoSONE*, 6(11), e27344. https://doi.org/10.1371journal.pone.0027344

Cordoni, G., & Palagi, E. (2012). Fair play and honest signals in immature chimpanzees. *Atti della Società Toscana di Scienze Naturali Serie B*, 119, 97-101. https://doi.org/10.2424/ASTSN.M.2012.14

Cordoni, G., Ciarcelluti, G., Pasqualotto, A., Perri, A., Bissiato, V., & Norscia, I. (2023). Is it for real? Structural differences between play and real fighting in adult chimpanzees (*Pan troglodytes*). *American Journal of Primatology*, Article e23537. https://doi.org/10.1002/ajp.23537

Cordoni, G., Gioia, M., Demuru, E., Norscia, I. (2021). The dark side of play: play fighting as a substitute for real fighting in domestic pigs (*Sus scrofa*). *Animal Behaviour,* 175, 21-31. https://doi.org/10.1016/j.anbehav.2021.02.016

Cordoni, G., Nicotra, V., & Palagi, E. (2016). Unveiling the ‘secret’ of dog play success: asymmetry and signals. *Journal of Comparative Psychology*, 130, 278-287. https://doi.org/10.1037/com0000035.

Cordoni, G., Norscia, I., Bobbio, M., & Palagi, E. (2018). Differences in play can illuminate differences in affiliation: a comparative study on chimpanzees and gorillas. *PLoSONE*, 13(3), e0193096. https://doi.org/10.1371/journal. pone.0193096.

Cordoni, G., Pirarba, L., Elies, S., Demuru, E., Guéry, J. P., & Norscia I. (2022). Adult–adult play in captive lowland gorillas (*Gorilla gorilla gorilla*). *Primates,* 63(3), 225-235. https://doi.org/10.1007/s10329-022-00973-7

Correia-Caeiro, C., Costa, R., Hayashi, M., Burrows, A., Pater, J., Miyabe-Nishiwaki, T., et al. (2025). GorillaFACS: the Facial Action Coding System for the Gorilla spp. *PLoS ONE*, 20(1), e0308790. https://doi.org/10.1371/journal.pone.0308790

Davila-Ross, M., & Dezecache, G. (2021). The complexity and phylogenetic continuity of laughter and smiles in hominids. *Frontiers in Psychology*, 12, 2065. https://doi.org/10.3389/ fpsyg.2021.648497

Davila-Ross, M., & Palagi, E. (2022). Laughter, play faces and mimicry in animals: evolution and social functions. *Philosophical Transaction Royal Society B*, 377, 20210177. https://doi.org/10.1098/rstb.2021.0177

Davila-Ross, M., & Zimmermann, E. (2009). *Towards the evolution of laughter: a comparative analysis on hominoids*. Saarbrücken, Germany: Südwestdeutscher Verlag für Hochschulschriften.

Davila-Ross, M., Allcock, B., Thomas, C., & Bard, K. A. (2011). Aping expressions? Chimpanzees produce distinct laugh types when responding to laughter of others. *Emotion*, 11, 1013-1120. https://doi.org/10.1037/a0022594

Davila-Ross, M., Jesus, G., Osborne, J, & Bard, K. A. (2015). Chimpanzees (*Pan troglodytes*) produce the same types of ‘laugh faces’ when they emit laughter and when they are silent. *PLoSONE*, 10, e0127337. https://doi.org/10.1371/journal.pone.0127337

Davila-Ross, M., Menzler, S., & Zimmermann, E., (2008). Rapid facial mimicry in orangutan play. *Biology Letters*, 4(1), 27-30. https://doi.org/10.1098/rsbl.2007.0535

de Waal, F. B. M., & Preston, S.D. (2017). Mammalian empathy: Behavioural manifestations and neural basis. *Nature Reviews Neuroscience,* 18*,* 498-509. https://doi.org/10.1038/nrn.2017.72

Demuru, E., Ferrari, P. F., & Palagi, E. (2015). Emotionality and intentionality in bonobo playful communication. *Animal Cognition,* 18, 333-344. https://doi.org/10.1007/s10071-014-0804-6

Dezecache, G., & Dunbar, R. I. (2012). Sharing the joke: the size of natural laughter groups. *Evolution and Human Behavior*, 33, 775-779. https://doi.org/10.1016/j.evolhumbehav.2012.07.002

Dimberg, U., & Thunberg, M. (1998). Rapid facial reactions to emotional facial expressions. *Scandinavian Journal of Psychology*, 39, 39-45. https://doi.org/10.1111/1467-9450.00054

Diogo R., Wood, B. A.,  Aziz, M. A., & Burrows A. (2009). On the origin, homologies and evolution of primate facial muscles, with a particular focus on hominoids and a suggested unifying nomenclature for the facial muscles of the Mammalia. *Journal of Anatomy*, 215(3), 300-319 https://doi.org/10.1111/j.1469-7580.2009.01111.x

Diogo, R., Potau, J. M., Pastor, J. F., de Paz, F. J., Ferrero, E. M., Bello, G., Barbosa, M., & Wood, B. A. (2010). *Photographic and descriptive musculoskeletal atlas of Gorilla, with notes on the attachments, variations, innervation, synonymy and weight of the muscles*. Enfield: Science Publishers.

Drack, P., Huber, T., & Ruch, W. (2009). The apex of happy laughter: a FACSstudy with actors. In E. Bänninger-Huber, & D. Peham (Eds.), *Current and future perspectives in facial expression research: topics and methodical questions* (pp. 32-37). Innsbruck University Press

Dunbar, R. I. M. (2022). Laughter and its role in the evolution of human social bonding. *Philosophical Transaction Royal Society B*, 377, 20210176. https://doi.org/10.1098/rstb.2021.0176

Ekman P., (2003). *Emotions revealed: recognizing faces and feelings to improve communication and emotional life*. New York, NY: Times Books/Henry Holt and Co.

Ekman, P., & Friesen, W. (1978). *Facial Action Coding System: a technique for the measurement of facial movement*. Consulting Psychologists Press

Ekman, P., Friesen, W. V., & Hager, J. C. (2002). *Facial Action Coding System*. Research Nexus

Fagen, R. (1981). *Animal play behavior*. Oxford University Press

Ferrari, P. F., Gallese, V., Rizzolatti, G., & Fogassi, L. (2003). Mirror neurons responding to the observation of ingestive and communicative mouth actions in the monkey ventral premotor cortex. *European Journal of Neuroscience,* 17, 1703-1714. 10.1046/j.1460-9568.2003.02601.x

Gallese, V., Fadiga, L., Fogassi, L., Rizzolatti, G. (1996). Action recognition in the premotor cortex. *Brain,* 119, 593-609. 10.1093/brain/119.2.593.

Gervais, M., & Wilson, D. S. (2005). The evolution and functions of laughter and humor: a synthetic approach. *Quarterly Review of Biology*, 80, 395-430. https://doi.org/10.1086/498281

Gilbert, M., Demarchi, S., & Urdapilleta, I. (2021). FACSHuman, a software program for creating experimental material by modeling 3D facial expressions. *Behavior Research Methods*, 53, 2252-2272.

Hess, U., & Fischer, A. (2013). Emotional mimicry as social regulation. *Personality and Social Psychological Review*, 17(2), 142-157. https://doi.org/10.1177/1088868312472607.

Iwase, M., Ouchi, Y., Okada, H., Yokoyama, C., Nobezawa, S., Yoshikawa, E., et al. (2002). Neural substrates of human facial expression of pleasant emotion induced by comic films: a PET study. *Neuroimage*, 17(2), 758-768. https://doi.org/10.1006/nimg.2002.1225

Jeganathan, J., Campbell, M., Hyett, M., Parker, G., & Breakspear, M. (2022). Quantifying dynamic facial expressions under naturalistic conditions. *Elife*, 11, e79581. https://doi.org/10.7554/eLife.79581

Kastendieck, T., Mauersberger, H., Blaison, C., Ghalib, J., & Hess, U. (2021). Laughing at funerals and frowning at weddings: top-down influences of context-driven social judgments on emotional mimicry. *Acta Psychologica*, 212, 103195. https://doi.org/10.1016/j.actpsy.2020.103195

Kavanagh, E., Whitehouse, J., & Waller, B. (2022). The face in everyday social interaction: social outcomes and personality correlates of facial behaviour. *Psyarxiv*. https://doi.org/10.31234/osf.io/7tbyr

Keylock, C. (2005). Simpson diversity and the Shannon-Wiener index as special cases of a generalized entropy. *Oikos*, 109, 203-207. https://doi.org/10.1111/j.0030-1299.2005.13735.x.

Mancini, G., Ferrari, P. F., & Palagi, E., (2013). Rapid facial mimicry in geladas. *Scientific* *Reports*, 3(1), 1-6. https://doi.org/10.1038/srep01527

Martin, R. A., & Ford, T. (2018). *The psychology of humor: an integrative approach*. New York Academic Press

Masai, K., Perusquía-Hernández, M., Sugimoto, M., Kumano, S., & Kimura, T. (2022). Consistent smile intensity estimation from wearable optical sensors. 10^th^ International Conference on Affective Computing and Intelligent Interaction (ACII). https://doi.org/10.1109/ACII55700.2022.9953867

Mascaró, M., Serón, F. J., Perales, F. J., Varona, J., & Mas, R. (2021). Laughter and smiling facial expression modelling for the generation of virtual affective behavior. *PlosOne*, 16*(5)*, e0251057. https://doi.org/10.1371/journal.pone.0251057

Masi, S., Cipolletta, C., & Robbins, M. M. (2009). Western lowland gorillas (*Gorilla gorilla gorilla*) change their activity patterns in response to frugivory. *American Journal of Primatology*, 71, 91-100. https://doi.org/10.1002/ajp.20629

Mehu, M., & Dunbar, R. I. (2008). Relationship between smiling and laughter in humans (*Homo sapiens*): testing the power asymmetry hypothesis. *Folia Primatologica*, 79(5), 269-280. https://doi.org/10.1159/000126928

Palagi, E., & Mancini, G. (2011). Play and primates: Social, communicative, and cognitive aspects of one of the most puzzling behaviour. *Atti della Società Toscana di Scienze Naturali Serie B,* 118*,* 121-128. https://doi.org/10.2424/ASTSN.M.2011.32

Palagi, E., Antonacci, D., & Cordoni, G. (2007). Fine-tuning of social play in juvenile lowland gorillas (*Gorilla gorilla gorilla*). *Developmental Psychobiology,* 49, 433-445. https://doi.org/10.1002/dev.20219

Palagi, E., Burghardt, G. M., Smuts, B., Cordoni G., Dall’Olio, S., Fouts, H. N., Řeháková-Petrů, M., & Pellis, S. M. (2016a) Rough-and-tumble play as a window on animal communication. *Biological Reviews,* 91, 311-327. http://dx.doi.org/10.1111/brv.12172

Palagi, E., Caruana, F., & de Waal F. B. M. (2022). The naturalistic approach to laughter in humans and other animals: towards a unified theory. *Philosophical Transaction Royal Society B*, 377, 20210175. https://doi.org/10.1098/rstb.2021.0175

Palagi, E., Celeghin, A., Tamietto, M., Winkielman, P., & Norscia, I. (2020). The neuroethology of spontaneous mimicry and emotional contagion in human and non-human animals. *Neuroscience and Biobehavioral Review*, 111, 149–165. https://doi.org/10.1016/j.neubiorev.2020.01.020

Palagi, E., Cordoni, G., Demuru, E., & Bekoff, M. (2016b). Fair play and its connection with social tolerance, reciprocity and the ethology of peace. *Behaviour,* 153, 1195*-*1216. http://dx.doi.org/10.1163/1568539X-00003336

Palagi, E., Marchi, E., Cavicchio, P., & Bandoli, F. (2019a). Sharing playful mood: rapid facial mimicry in *Suricata suricatta*. *Animal Cognition,* 22, 719-732. https://doi.org/10.1007/s10071-019-01269-y

Palagi, E., Nicotra, V., & Cordoni, G. (2015). Rapid mimicry and emotional contagion in domestic dogs. *Royal Society Open Science*, 2, 150505. https://doi.org/10.1098/rsos.150505.

Palagi, E., Norscia, I., Pressi, S., & Cordoni, G. (2019b). Facial mimicry and play: a comparative study in chimpanzees and gorillas. *Emotion*, 19*(4)*, 665-681. http://dx.doi.org/10.1037/emo0000476

Paquette, D. (1994). Fighting and playfighting in captive adolescent chimpanzees. *Aggressive Behavior*, 20(1), 49-65. https://doi.org/10.1002/1098-2337(1994)20:1<49::AID-AB2480200107>3.0.CO;2-C.

Parr, L. A., Waller, B. M., Burrows, A. M., Gothard, K. M., & Vick, S. J. (2010). Brief ommunication: MaqFACS: a muscle-based facial movement coding system for the rhesus macaque. *American Journal of Physical Anthropology*, 143, 625-630. https://doi.org/10.1002/ajpa.21401.

Parr, L. A., Waller, B. M., Vick, S. J., & Bard, K. A. (2007). Classifying chimpanzee facial expressions using muscle action. *Emotion,* 7*(1)*, 172-181. https://doi.org/10.1037/1528-3542.7.1.172

Pellis, S. M., & Pellis, V. C. (2017). What is play fighting and what is it good for? *Learning & behavior*, 45(4), 355-366. https://doi.org/10.3758/s13420-017-0264-3.

Pellis, S. M., Pellis, V. C., & Reinhart, C. J. (2010). The evolution of social play. In C. Worthman, P. Plotsky, D. Schechter, & C. Cummings (Eds.), *Formative Experiences: The Interaction of Caregiving, Culture, and Developmental Psychobiology* (pp. 404-431). Cambridge University Press.

Pellis. S. M., & Pellis, V. C. (1996). On knowing it’s only play: the role of play signals in play fighting. *Aggressive and Violent Behavior,* 1, 249-268. https://doi.org/10.1016/1359-1789(95)00016-X

Pellis. S. M., & Pellis, V. C. (1997). Targets, tactics and the open mouth face during play fighting in three species of primates. *Aggressive Behavior*, 23, 41-57. https://doi.org/10.1002/(SICI)1098-2337(1997)23:1<41::AID-AB5>3.0.CO;2-W

Porges, S. W. (2011). *The Polyvagal Theory*. W.W. Norton and Company

Preuschoft, S., & van Hooff, J. A. R. A. M. (1997). The social function of “smile” and “laugther”: variations across primate species and societies. In U. Segerstrale, & P. Molnàr (Eds.), *Nonverbal communication: Where nature meets culture* (pp. 171–189). Mahwah, NJ: Erlbaum

Provine, R. R. (1992). Contagious laughter: laughter is a sufficient stimulus for laughs and smiles. *Bulletin of the Psychonomic Society*, 30, 1-4. http://dx.doi.org/10.3758/BF03330380

Provine, R. R. (2000). *Laughter: a scientific investigation*. Viking Penguin

Redican, W. K. (1982). An evolutionary perspective on human facial displays. In P. Ekman (Ed.), *Emotion in the human face* (pp. 212-281). Cambridge University Press

Reed, D. D., & Azulay, R. L. (2010). A Microsoft Excel® 2010 based tool for calculating Interobserver Agreement. Behavior Analysis in Practice, 4(2), 45-52. http://dx.doi.org/10.1007/BF03391783

Ruch,W., & Ekman, P. (2001). The expressive pattern of laughter. In A. Kaszniak (Ed.), *Emotion, Qualia, and Consciousness* (pp. 426-443). Tokyo: World Scientific

Sarra, S., & Otta, E. (2001). Different types of smiles and laughter in preschool children. *Psychological Reports,* 89*,* 547-558. http://dx.doi.org/10.2466/pr0.2001.89.3.547

Scopa, C., & Palagi, E., (2016). Mimic me while playing! Social tolerance and rapid facial mimicry in macaques (*Macaca tonkeana* and *Macaca fuscata*). *Journal of Comparative Psychology*, 130(2), 153-161. http://dx.doi.org/10.1037/com0000028

Scott, S. K., Lavan, N., Chen, S., & McGettigan, C. (2014). The social life of laughter. *Trends in Cognitive Science*, 18, 618-620. https://doi.org/10.1016/j.tics.2014.09.002

Sestito, M., Umiltà, M. A., De Paola, G., Fortunati, R., Raballo, A., Leuci, E., & Gallese, V. (2013). Facial reactions in response to dynamic emotional stimuli in different modalities in patients suffering from schizophrenia: a behavioral and EMG study. *Frontiers in Human Neuroscience*, 7, 368. https://doi.org/10.3389/fnhum.2013.00368

Shannon, C. E. (1948). A mathematical theory of communication. *The Bell System Technical Journal*, 27(3), 379-423. https://doi.org/10.1002/j.1538-7305.1948.tb01338.x.

Shultz, S., & Dunbar, R. I. M. (2012). The social brain hypothesis: an evolutionary perspective on the neurobiology of social behaviour. *I Know What You Are Thinking: Brain Imaging and Mental Privacy*, 13-28.

Simons, D. J., Shoda, Y., & Lindsay, D. S. (2017). Constraints on Generality (COG): a proposed addition to all empirical papers. *Perspectives on Psychological Science*, 12(6),1123-1128. https://doi.org/10.1177/1745691617708630

Thierry, B., Demaria, C., Preuschoft, S., & Desportes, C. (1989). Structural convergence between silent baredteeth display and relaxed open-mouth display in the Tonkean macaque (*Macaca tonkeana*). *Folia Primatologica*, 52, 178-184. https://doi.org/10.1159/000156396

Thompson, K. V. (1998). Self assessment in juvenile play. In M. Bekoff M, & J. A. Byers (Eds), *Animal Play: Evolutionary, Comparative and Ecological Perspective* (pp. 183-204). Cambridge University Press

Tinbergen, N. (1952). "Derived" activities; their causation, biological significance, origin, and emancipation during evolution. *Quarterly Review of Biology*, 27(1), 1-32.

van Hooff, J. A. R. A. M., & Preuschoft, S. (2003). Laughter and smiling: the intertwining of nature and culture. In F.B.M. de Waal, & P.L. Tyack (Eds.), *Animal social complexity: intelligence, culture, and individualized societies* (pp. 261-287). Harvard University Press

Vick, S. J., Waller, B. M., Parr, L. A., Pasqualini, M. C. S., & Bard, K. A. (2007). A cross-species comparison of facial morphology and movement in humans and chimpanzees using the Facial Action Coding System (FACS). *Journal of Nonverbal Behav*ior, 31, 1-20. https://doi.org/10.1007/s10919-006-0017-z

Waller, B. M., & Cherry, L. (2012). Facilitating play through communication: significance of teeth exposure in the gorilla play face. *American Journal of Primatology*, 74, 157-164.  https://doi.org/10.1002/ajp.21018

Waller, B. M., & Micheletta, J. (2013). Facial expression in nonhuman animals. *Emotion Review*, 5, 54-59. https://doi.org/10.1177/1754073912451503.

Waller, B. M., Caeiro, C. C., & Davila-Ross, M. (2015). Orangutans modify facial displays depending on recipient attention. *PeerJ*, 3, e827. https://doi.org/10.7717/peerj.827.

Waller, B. M., Julle-Daniere, E., & Micheletta, J. (2020). Measuring the evolution of facial 'expression' using multi-species FACS. *Neuroscience and Biobehavioral Reviews*, 113, 1-11. https://doi.org/10.1016/j.neubiorev.2020.02.031

Waller, B. M., Lembeck, M., Kuchenbuch, P., Burrows, A. M., & Liebal, K. (2012). GibbonFACS: a muscle-based facial movement coding system for hylobatids. *International Journal of Primatology*, 33, 809-821. https://doi.org/10.1007/s10764-012-9611-6.

Waller, B. M., Parr, L. A., Gothard, K. M., Burrows, A. M., & Fuglevand, A. J. (2008). Mapping the contribution of single muscles to facial movements in the rhesus macaque. *Physiology and Behavior*, 95, 93-100. https://doi.org/10.1016/j.physbeh.2008.05.002.

Waller, B.M., Bard, K.A., Vick, S.J., & Pasqualini, M.C.S. (2007). Perceived differences between chimpanzee (*Pan troglodytes*) and human (*Homo sapiens*) facial expressions are related to emotional interpretation. *Journal of Comparative Psychology*, 121, 398–404. https://doi.org/10.1037/0735-7036.121.4.398.

Wathan, J., Burrows, A. M., Waller, B. M., & McComb, K. (2015). EquiFACS: the equine facial action coding system. *PLoSOne*, 10, e0131738. https://doi.org/10.1371/journal.pone.0131738.

Weisfeld, G. E. (1993). The adaptive value of humor and laughter. *Ethology and Sociobiology,* 14*,* 141-169. http://dx.doi.org/10.1016/0162-3095(93)90012-7

Wood, A., & Niedenthal, P. (2018). Developing a social functional account of laughter. *Social and Personality Psychology Compass*, 12, e12383. https://doi.org/10.1111/spc3.12383

**FIGURE CAPTIONS**

**Figure 1 -** An example of image capturing with OpenFace 2.0. In the three images (neutral face - N, play face - PF, and full play face - FPF) 3D facial landmarks (red/blue dots), head pose traking (blue/violet 3D polygons) and eye gaze traking (light green lines) are represented.

**Figure 2 -** Graphical representation of the individual perception condition. In the *yes-perception* condition the first stimulus by the trigger fell into frontal or lateral view of the potential receiver. In the *no-perception* condition the stimulus fell into the blind area of the potential receiver.

**Figure 3 -** Violin plots with included boxplots representing A.) the levels of play face (PF) and full play face (FPF) calculated as the total number of PFs/FPFs performed by an individual divided by total number of play sessions in which this individual was involved, and B.) the median duration values (in sec) of both PF and FPF performed by each individual. The shape of the violin represents the density estimate of the variable considered: the more data points in a specific range the larger the violin shape is for that range. Dots represent the individual data points (N_individuals_=21). In the middle of each density curve, there is a small boxplot with the rectangle showing the ends of the first and third quartiles and the central line showing the median value.

**Figure 4 -** Error bars representing A.) the number of PFs replicated (=1) and not replicated (=0) by potential responders within 1 sec after the emission of the first PFs by triggers under the two perception conditions: PF perceived (=1) and PF not-perceived (=0); number of PF perceived and replicated = 33; number of PF perceived and not replicated = 44; number of PF not perceived and replicated = 8; number of PF not perceived and not replicated = 32; and B.) the number of FPFs replicated (=1) and not replicated (=0) by potential responders within 1 sec after the emission of the first FPFs by triggers under the two perception conditions: FPF perceived (=1) and FPF not-perceived (=0); number of FPF perceived and replicated = 171; number of FPF perceived and not replicated = 96; number of FPF not perceived and replicated = 15; number of FPF not perceived and not replicated = 61. The length of bars indicates how spread the data are around the mean value (colored dots).

**Figure 5 -** Violin plot with included boxplots representing the time period (in sec) between the occurrence of the first playful expression or RFM event and the end of the play session across five different conditions: i) players performed at least two not-mimicked PFs only (*PF-not-mim*); ii) players performed at least two not-mimicked FPFs only (*FPF-not-mim*); iii) at least one event of RFM of PF (but not FPF) occurred (*PF_RFM*); iv) at least one event of RFM of FPF (but not PF) occurred (*FPF_RFM*), and v) at least one event of both RFM of PF and RFM of FPF occurred in the same play session (*PF&FPF_RFM*). The shape of the violin represents the density estimate of the variable considered. Dots represent the individual data points (N_dyads_PF-not-mim_=36, N_dyads_FPF-not-mim_=56, N_dyads_PF_RFM_=20, N_dyads_FPF_RFM_ =67, N_dyads_PF&FPF_RFM_=11). The rectangle of the small boxplot shows the ends of the first and third quartiles and the central line shows the median value. Only probabilities of significant results are representing in the graph.

**Figure 6 -** Error bars representing A.) the mean values of the Play Asymmetry Index (PAI) calculated before and after the performance of a not mimicked PF (*PF-not-mim*), not mimicked FPF (*FPF-not-mim*) and an event of PF mimicry (*PF_RFM*) and FPF mimicry (*FPF_RFM*), and B.) the mean values of the Shannon index (H’) calculated before and after the performance of a not mimicked PF (*PF-not-mim*), not mimicked FPF (*FPF-not-mim*) and an event of PF mimicry (*PF_RFM*) and FPF mimicry (*FPF_RFM*). The length of bars indicates how spread the data are around the mean value (colored dots). Only probabilities of significant results are representing in the graphs. The sample size of each condition is indicated on the graph.
