# Supplementary figures and images for: Functional and Morphological Differences in the Play Face and Full Play Face in Lowland Gorillas, a Hominid Species: Implications for the Evolutionary Roots of Smile and Laugh Face
Source: Am J Biol Anthropol. 2025 May 8;187(1):e70061. doi: 10.1002/ajpa.70061 (PMC12062928; doi:10.1002/ajpa.70061)

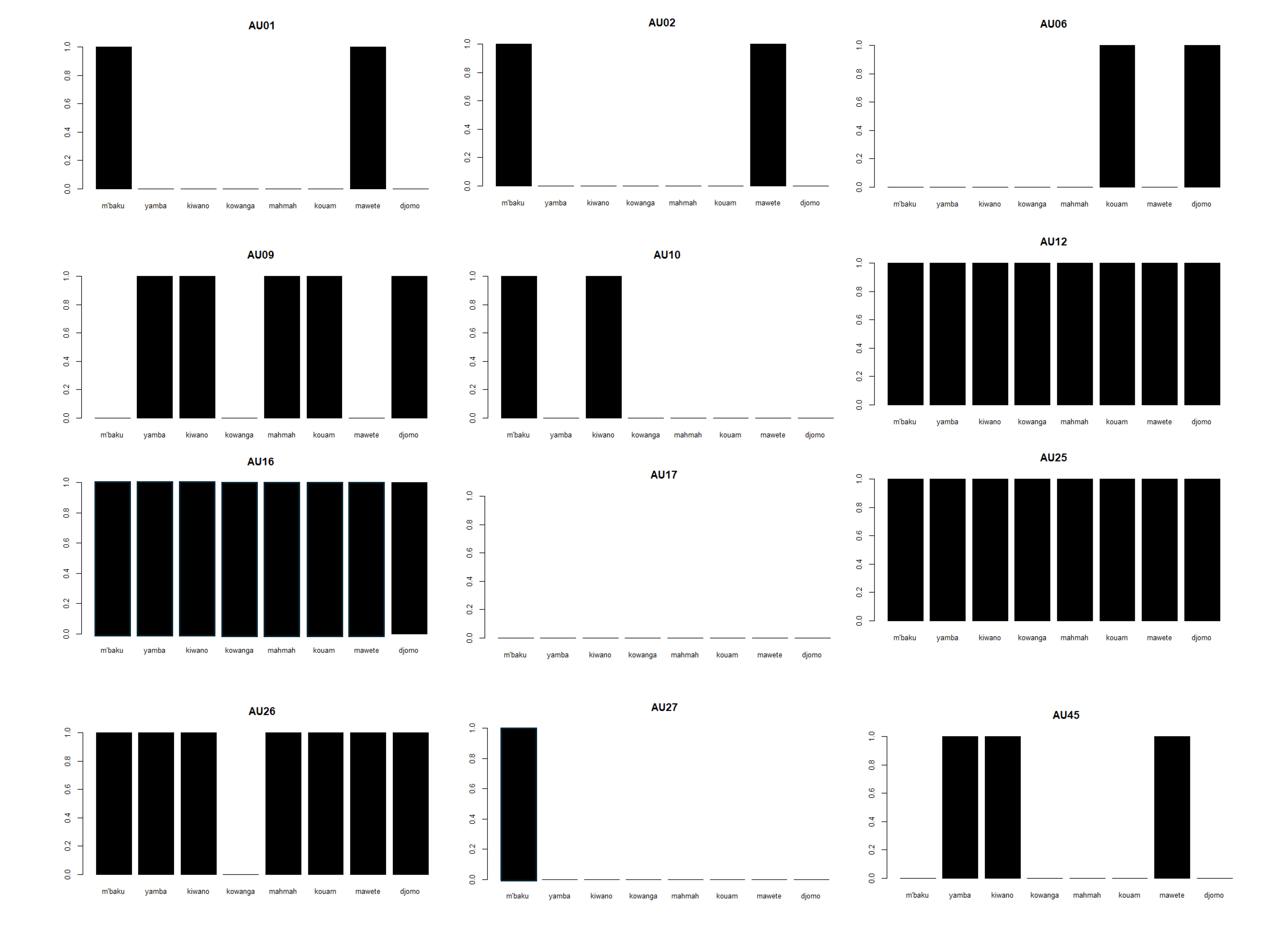

Supplement: Supplementary file 1 — Data S1. [file AJPA-187-e70061-s001.zip › R3_AJBA_play_face_full_play_face_gorillas/Figure_S3_graphs_Action_Unit_activation_play_face_tif.tif]

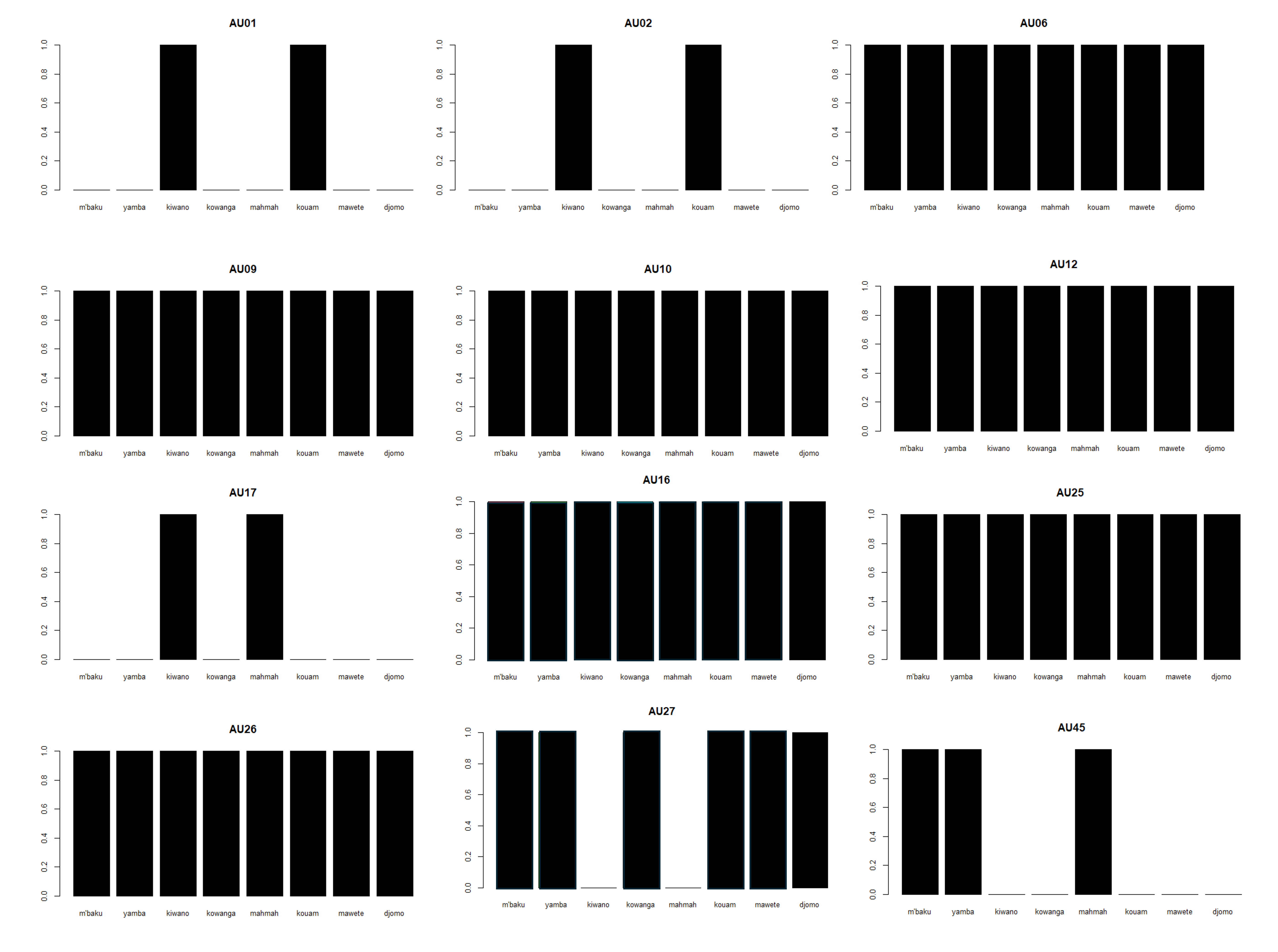

Supplement: Supplementary file 1 — Data S1. [file AJPA-187-e70061-s001.zip › R3_AJBA_play_face_full_play_face_gorillas/Figure_S4_graphs_Action_Unit_activation_full_play_face_tif.tif]
