# Supplementary material for: Functional and Morphological Differences in the Play Face and Full Play Face in Lowland Gorillas, a Hominid Species: Implications for the Evolutionary Roots of Smile and Laugh Face
Source: Am J Biol Anthropol. 2025 May 8;187(1):e70061. doi: 10.1002/ajpa.70061 (PMC12062928; doi:10.1002/ajpa.70061)
Supplement: Supplementary file 1 — Data S1. [file AJPA-187-e70061-s001.zip › R3_AJBA_play_face_full_play_face_gorillas/R3_AJBA_Play_face_full_play_face_in_gorillas_COVER_LETTER.docx]

Dear Editor,

first of all, we would like to thank you for your crucial support in the publication of our manuscript.

As requested by Reviewer 1, we have mentioned in our manuscript that GorillaFACS has now been published, although it was not yet available at the time of our study.

Thank you once again.

Best regards,

The Authors
