# Supplementary material for: Functional and Morphological Differences in the Play Face and Full Play Face in Lowland Gorillas, a Hominid Species: Implications for the Evolutionary Roots of Smile and Laugh Face
Source: Am J Biol Anthropol. 2025 May 8;187(1):e70061. doi: 10.1002/ajpa.70061 (PMC12062928; doi:10.1002/ajpa.70061)
Supplement: Supplementary file 1 — Data S1. [file AJPA-187-e70061-s001.zip › R3_AJBA_play_face_full_play_face_gorillas/R3_AJBA_Play_face_full_play_face_in_gorillas_REBUTTAL_LETTER.docx]

**Reviewer: 1**

Comments to the Author

I have no further suggestions compared with the response to my previous review.

One note in response to comments to the second reviewer; I notice much of the discussion with R2 revolves around the methodological aspects of FACS, some of which is due to a lack of GorillaFACS. In between these revisions, I see that GorillaFACS has now been published and appears publically available. Although I think suggesting a full recoding of videos would not be appropriate, I would hope authors now comment on this system in their MS (and hopefully, conclude the differences between Chimp and GorillaFACS are minimal).

Correia-Caeiro, C., Costa, R., Hayashi, M., Burrows, A., Pater, J., Miyabe-Nishiwaki, T., ... & Liebal, K. (2025). GorillaFACS: The Facial Action Coding System for the Gorilla spp. PloS one, 20(1), e0308790.

**ANSWER: thank you for your suggestion and support. We mentioned in different parts of the MS that during our study GorillaFACS was not yet available and we cited the relative publication (Correia-Caeiro et al., 2025). Furthermore, we already re-analyzed all PFs and FPFs with GorillaFACS as well and we obtained almost the same results found with the other tools.**
